# Five Novel Taxa from Freshwater Habitats and New Taxonomic Insights of Pleurotheciales and Savoryellomycetidae

**DOI:** 10.3390/jof7090711

**Published:** 2021-08-30

**Authors:** Wei Dong, Rajesh Jeewon, Kevin D. Hyde, Er-Fu Yang, Huang Zhang, Xiandong Yu, Gennuo Wang, Nakarin Suwannarach, Mingkwan Doilom, Zhangyong Dong

**Affiliations:** 1 Innovative Institute for Plant Health, Zhongkai University of Agriculture and Engineering, Guangzhou 510225, China; dongwei0312@hotmail.com (W.D.); kdhyde3@gmail.com (K.D.H.); j_hammochi@hotmail.com (M.D.); dongzhangyong@hotmail.com (Z.D.); 2 Center of Excellence for Fungal Research, Mae Fah Luang University, Chiang Rai 57100, Thailand; 3 Research Center of Microbial Diversity and Sustainable Utilization, Faculty of Science, Chiang Mai University, Chiang Mai 50200, Thailand; 4 Department of Biology, Faculty of Science, Chiang Mai University, Chiang Mai 50200, Thailand; 5 Department of Health Sciences, Faculty of Medicine and Health Sciences, University of Mauritius, Reduit 80837, Mauritius; r.jeewon@uom.ac.mu; 6 Research Center in Bioresources for Agriculture, Industry and Medicine, Chiang Mai University, Chiang Mai 50200, Thailand; erfu20170431@gmail.com; 7 Faculty of Agriculture and Food, Kunming University of Science & Technology, Kunming 650500, China; zhanghuang2002113@gmail.com; 8 School of Life Science and Technology, University of Electronic Science and Technology of China, Chengdu 611731, China; yuxiandong@std.uestc.edu.cn; 9 Faculty of Environmental Science and Engineering, Kunming University of Science & Technology, Kunming 650500, China; gennuo2021@gmail.com

**Keywords:** annulatascales-like, multi-locus phylogeny, *Pseudocoleodictyospora*, Sordariomycetes, submerged wood, Thailand, Yunnan

## Abstract

Pleurotheciales is the largest order in Savoryellomycetidae with a large proportion of species known from freshwater habitats. In order to investigate the phylogenetic relationships of taxa within Pleurotheciales and contribute to their diversity, submerged wood was collected from freshwater habitats in China (Yunnan Province) and Thailand. Two dematiaceous, sporodochial hyphomycetes and one annulatascales-like ascomycete with unusual morphology as compared to extant ones were discovered. They were subjected to DNA-based phylogenetic analyses and the results revealed three distinct lineages in Savoryellomycetidae. This morpho-phylo taxonomic study supports the establishment of five novel taxa including two novel genera, *Obliquifusoideum* and *Saprodesmium*, and three novel species, *Coleodictyospora muriformis*, *Obliquifusoideum guttulatum* and *Saprodesmium dematiosporum*. *Coleodictyospora muriformis* and *S. dematiosporum* are placed in Pleurotheciales, while *O. guttulatum* is referred to Savoryellomycetidae genera *incertae sedis*. The phylogenetic relationships are also presented for *Coleodictyospora* and *Pseudocoleodictyospora*, which raises an intriguing taxonomic issue. These two genera are positioned in two different classes, viz Sordariomycetes and Dothideomycetes, although they are quite similar except for the presence of a conidial sheath. This study expands our knowledge of the fungal diversity of freshwater fungi, and also indicates that Pleurotheciales species are mostly found in freshwater habitats.

## 1. Introduction

Savoryellomycetidae currently accommodates four orders, Conioscyphales, Fuscosporellales, Pleurotheciales and Savoryellales. This is based on evidence from phylogenetic analyses and divergence time studies with the order having a stem age estimated as 268 MYA [1]. The four orders clustered as a robust clade in all studies [1,2,3]. Pleurotheciales, with a single-family Pleurotheciaceae [4], is the largest order in Savoryellomycetidae. Pleurotheciaceae species have mostly been isolated from decaying wood or plant debris as saprobes, while few species were also identified as opportunistic human pathogens (*Phaeoisaria clematidis*) [5]. Multi-locus phylogenetic relationships of Pleurotheciaceae species were investigated to better understand their taxonomy [3,4,6,7,8,9,10] and as a result, eleven genera were accepted in the family [11].

Taxa of Pleurotheciaceae have perithecial ascomata with asexual dematiaceous hyphomycetous stages. Coelomyceteous asexual morphs have not been reported in the family. Pleurotheciaceae is an assemblage of genera representing a highly diverse morphology, especially in the asexual morphs. *Pleurotheciella* and *Pleurothecium* (type) generally have macronematous, unbranched conidiophores, holoblastic, sympodially proliferating conidiogenous cells with a conspicuous rachis of denticles, and hyaline, septate conidia [4,9,10,12,13]. *Anapleurothecium* has macronematous, unbranched conidiophores, sympodial, denticulate conidiogenous cells which are the cases of *Pleurotheciella* and *Pleurothecium*, but it has botuliform to cylindrical and brown conidia with a paler basal cell [7]. *Phaeoisaria* has synnematous conidiophores with tiny aseptate conidia [13]. *Phragmocephala* also has synnematous conidiophores, but the conidia are relatively large, ellipsoidal to subglobose, with dark brown to black central cells and paler polar cells [14]. *Sterigmatobotrys* are distinct in the family by their well-defined stipe and a complex penicillate conidiophore head consisting of series of penicillate branches [6]. While some other genera lack conspicuous macronematous conidiophores, and the conidia directly arise from the hyphae on the host substrate or from micronematous, subhyaline conidiophores, such as *Neomonodictys* with subglobose to globose, muriform conidia [8] and *Helicoascotaiwania* with helicoid conidia [2,15].

The diversity of morphology is also reflected in some sexual morphs of Pleurotheciaceae. The genera *Adelosphaeria*, *Melanotrigonum*, *Pleurotheciella* and *Pleurothecium* generally have superficial ascomata with a short papilla, narrowly or broadly clavate asci with a distinct, refractive apical ring and ellipsoidal-fusiform, septate ascospores [3,4,9], while *Phaeoisaria* has immersed ascomata with a quite long neck, cylindrical asci and filiform, multiseptate ascospores. *Helicoascotaiwania* is easily distinguished in the family in having generally immersed ascomata lying horizontally or obliquely to the host substrate and fusiform, versicolorous ascospores with darker central cells and paler polar cells [2,3].

The asexual–sexual morph connections were investigated based on cultural studies with a combination of molecular data. Some hyphomycetes were linked as the life cycle of known sexual morphs. The asexual morph of *Pleurothecium recurvatum* was first reported from the artificial medium (WA) inoculated by an ascomycetous species *Carpoligna pleurothecii* [16]. Réblová et al. [9] also found the asexual morphs from another medium (PCA) inoculated by ascomycetous species *Pleurotheciella rivularia* and *Pleurothecium semifecundum*. With DNA sequence data, Luo et al. [10] linked the asexual–sexual morph of *Pleurotheciella fusiformis* based on two specimens collected from Erhai Lake, Yunnan, China.

Luo et al. [6] used multi-locus analysis to first report a sexual morph in *Phaeoisaria*, namely *Ph. filiformis*, which was characterized by immersed ascomata with a long, cylindrical neck, and cylindrical asci containing hyaline and filiform ascospores. Although the morphological traits associate *Ph. filiformis* as allied with *Ceratosphaeria* and *Ophioceras* in Magnaporthales, phylogenetic analysis placed *Ph. filiformis* in Pleurotheciales. Neither conidia nor conidiomatal structures were observed from the culture of *Ph. filiformis* [6].

The main objectives of this study were to revise the taxon diversity within Pleurotheciales, report on novel taxa and provide new insights into the systematics of Savoryellomycetidae. Two specimens of dematiaceous hyphomycetes were preliminarily identified as Pleurotheciales species with micronematous conidiophores, holoblastic conidiogenous cells and dark muriform conidia, but the morphologies were rather unusual as compared to other members of the family. One specimen resembled annulatascaceae-like taxa in Diaporthomycetidae but is similar to the taxa of Pleurotheciaceae in Savoryellomycetidae. In order to clarify the placement of these specimens, a multi-locus analysis of a concatenated nuc 28S rDNA (LSU), nuc18S rDNA (SSU), internal transcribed spacer (ITS) and second-largest subunit of RNA polymerase II (*rpb2*) dataset were performed, and phylogenetic relationships inferred.

## 2. Materials and Methods

### 2.1. Herbarium Material, Isolation and Morphology

Decayed woody twigs and branches submerged in freshwater streams in forests were randomly collected in Yunnan Province, China, as well as Satun and Songkhla provinces in Thailand where all places are in the Greater Mekong Subregion. Specimens were placed in zip-lock plastic bags containing moistened cotton and taken to the laboratory. Sediment on separated specimens was washed off with tap water and incubated in plastic boxes lined with moistened tissue paper at room temperature (20–25 °C) for 1–2 weeks. The ascomata and sporodochia developed on the specimens were examined with a Nikon SMZ-171 dissecting microscope. Fungal structures were captured with a Nikon ECLIPSE Ni compound microscope fitted with a Canon EOS 750D digital camera. Single spore isolations were made from ascospore or conidium on potato dextrose agar (PDA, Shanghai Bio-way technology Co., Ltd., Shanghai, China) at room temperature (20–25 °C). All morphological approaches used herein were modified from Chomnunti et al. [17] and Senanayake et al. [18]. Tarosoft (R) Image Frame Work program was used to measure the fungal structures. Images were processed with Adobe Photoshop CS5 software (Adobe Systems, San Jose, CA, USA). Herbarium specimens (dry wood with fungal materials) were deposited in the herbarium of Mae Fah Luang University (MFLU), Chiang Rai, Thailand and herbarium of Cryptogams, Kunming Institute of Botany Academia Sinica (HKAS), Kunming, China. Living cultures were deposited in the Mae Fah Luang University Culture Collection (MFLUCC) and Kunming Institute of Botany Culture Collection (KUMCC). The novel taxa were registered in the databases Facesoffungi (http://www.facesoffungi.org, accessed on 10 June 2021) [19] and Index Fungorum (http://www.indexfungorum.org/names/names.asp, accessed on 21 June 2021).

### 2.2. DNA Extraction, PCR Amplification and Sequencing

Fungal mycelia were scraped from the colonies on PDA. The Biospin Fungus Genomic DNA Extraction Kit (Bioer Technology Co., Hangzhou, China) was used to extract total genomic DNA. The polymerase chain reaction (PCR) technique was utilized for the amplification of target DNA fragments. The primer pairs LR0R/LR5, NS1/NS4, ITS5/ITS4 and fRPB2-5F/fRPB2-7cR were used to amplify LSU, SSU, ITS and *rpb2* [20,21,22]. The amplifications were carried out as detailed in Dong et al. [23]. The PCR thermal cycle program for the amplification of LSU, SSU and ITS was provided as initially 94 °C for 3 min, followed by 35 cycles of denaturation at 94 °C for 30 s, annealing at 55 °C for 50 s, elongation at 72 °C for 90 s and a final extension at 72 °C for 10 min. The annealing was adjusted to 52 °C for *rpb2.* PCR products were checked on 1% agarose electrophoresis gels stained with Gel Red. The sequencing reactions were carried out by Shanghai Sangon Biological Engineering Technology and Services Co., Shanghai, China.

### 2.3. Molecular Phylogenetic Analyses

#### 2.3.1. Sequence Selection and Phylogenetic Analyses Construction

The qualities of generated sequences were initially checked with Finch TV v. 1.4.0 and each gene was subjected to a BlastN search in NCBI’s GenBank to assess the confidence level. Phylogenetic placements of the unidentified fungi were resolved by analyzing four different datasets: (1) two multi-locus datasets of a concatenated LSU, SSU, ITS and *rpb2* sequences; (2) two separate individual LSU and ITS datasets. The first multi-locus dataset was analysed to infer the phylogenetic positions of unidentified fungi within the Savoryellomycetidae along with sequences deposited from recent relevant publications [3,6,24]. In the second multi-locus dataset, we included the other subclasses in Sordariomycetes to infer the subclass status of an unidentified genus which could not be confirmed in any orders in the subclass Savoryellomycetidae. Besides, *Pseudocoleodictyospora* and its related taxa were also included to show the phylogenetic relationships between *Coleodictyospora* and *Pseudocoleodictyospora*. The individual LSU and ITS phylogenetic analyses were utilized to auxiliarily assess the phylogenetic relationships of fungi in Savoryellomycetidae. All sequences used in this study were listed in Table 1.

#### 2.3.2. Maximum Likelihood Analyses

Each dataset was aligned with MAFFT v. 7.409 online version [25] and manually verified with BioEdit v. 7.2.5 Biological Sequence Alignment Editor (Ibis BioSciences, Carlsbad, CA, USA), and then concatenated with Mesquite v. 3.11. The maximum likelihood (ML) analyses were performed with RAxML-HPC v. 8 on XSEDE in CIPRES Science Gateway [26,27], with the following changes from the default settings: maximum hours to run: 5; model for bootstrapping phase: GTRGAMMA; analysis type: rapid bootstrap analysis/search for best-scoring ML tree (-f a); bootstrapping type: rapid bootstrapping (-x); bootstrap iterations: 1000 (the maximum value allowed).

#### 2.3.3. Bayesian Inference Analyses

The Bayesian inference (BI) analyses were performed with MrBayes on XSEDE also in CIPRES Science Gateway [26,27]. In the first analysis of Savoryellomycetidae, the best-fit model was GTR+I+G for LSU, ITS and *rpb2*, and SYM+I+G for SSU. Six simultaneous Markov chains were run for 965,100 generations and trees were sampled every 100th generation. In total, 9651 trees were sampled and the first 25% of sampled trees representing the burn-in phase of the analyses were discarded and the remaining 7239 trees were used for calculating posterior probabilities (PP) in the majority rule consensus tree (critical value for the topological convergence diagnostic is 0.01) [28].

In the second analysis, the best-fit model was GTR+I+G for all datasets. Six simultaneous Markov chains were run for 685,100 generations and trees were sampled every 100th generation. In total, 6851 trees were sampled and the first 25% of sampled trees representing the burn-in phase of the analyses were discarded and the remaining 5139 trees were used for calculating posterior probabilities (PP) in the majority rule consensus tree (critical value for the topological convergence diagnostic is 0.01) [28].

Phylogenetic trees were viewed with FigTree v. 1.4.03 (http://tree.bio.ed.ac.uk/ accessed on 5 May 2021) and edited with Microsoft Office PowerPoint 2007 (Microsoft Corporation, WA, USA).

## 3. Results

### 3.1. Phylogenetic Analyses

**Figure 1:**
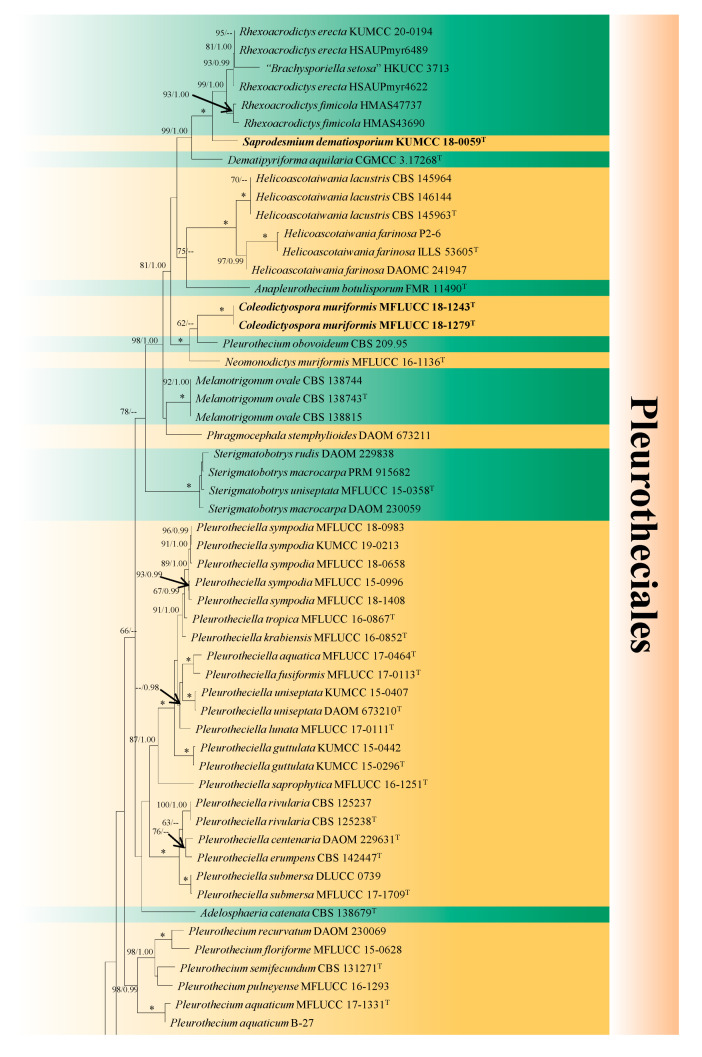

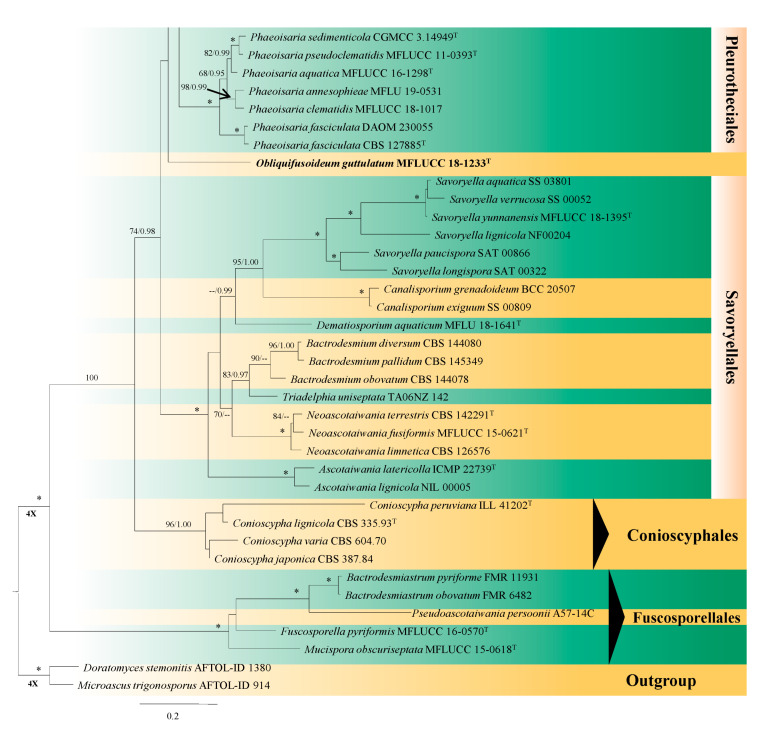
RAxML tree of Savoryellomycetidae with four orders, Conioscyphales, Fuscosporellales, Pleurotheciales and Savoryellales. The multi-locus tree is generated from combined LSU, SSU, ITS and *rpb2* sequence data. Bootstrap support values for maximum likelihood (the first value) equal to or greater than 60% and Bayesian posterior probabilities (the second value) equal to or greater than 0.95 are placed near the branches as ML BS/BI PP. The asterisk “*” represents bootstrap support values with 100% ML BS and 1.00 BI PP. The tree is rooted to *Doratomyces stemonitis* AFTOL-ID 1380 and *Microascus trigonosporus* AFTOL-ID 914. The ex-type cultures are indicated using “^T^” after strain numbers and the new species introduced in this study are indicated in **bold**.

In the first phylogenetic analysis (Figure 1), the representative homologous sequences of Conioscyphales, Fuscosporellales and Savoryellales and sequences from all genera of Pleurotheciales representing 90 isolates and two outgroup taxa (*Doratomyces* *stemonitis* AFTOL-ID 1380 and *Microascus trigonosporus* AFTOL-ID 914) were included. The matrix had 2205 distinct alignment patterns, with 41.06% of completely undetermined characters and gaps. In the RAxML tree, three distinct independent lineages were identified: (1) one new genus *Obliquifusoideum* (no bootstrap support); (2) one new genus *Saprodesmium* (100% ML BS/1.00 BI PP); (3) *Coleodictyospora* (62% ML BS/--) with one new species *C. muriformis*.

**Figure 2:**
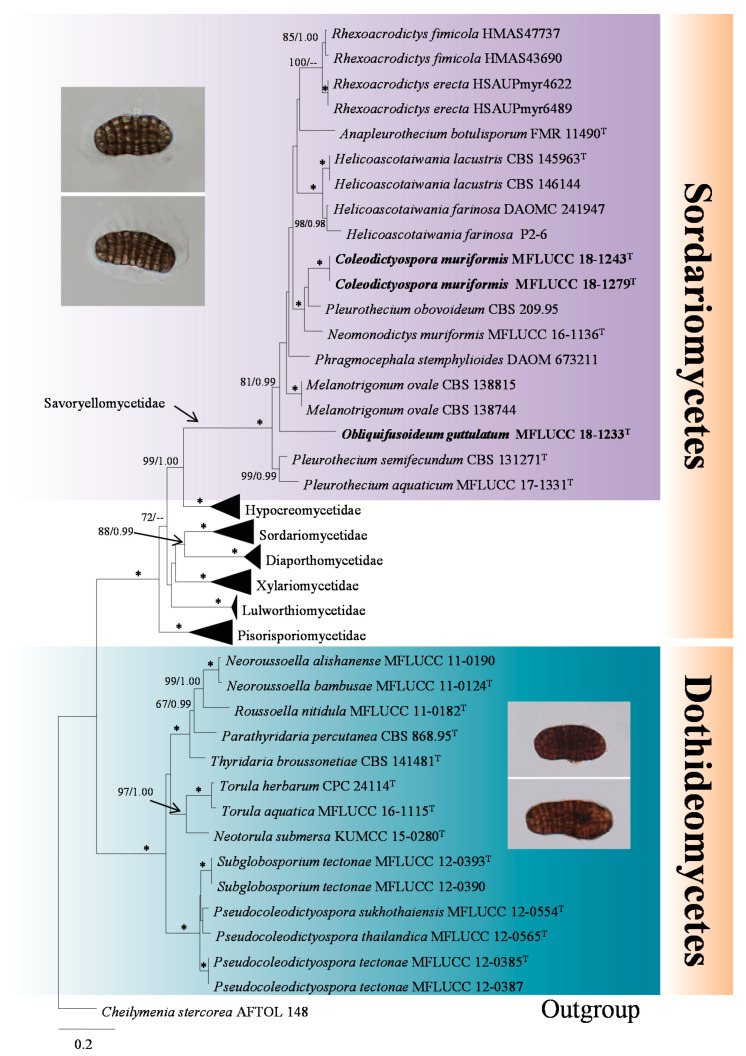
RAxML tree with taxa from two classes, Dothideomycetes and Sordariomycetes, to show the phylogenetic relationships between *Coleodictyospora* and *Pseudocoleodictyospora*. The illustrations of species in *Coleodictyospora* and *Pseudocoleodictyospora* are displayed near the generic names. The multi-locus tree is generated from combined LSU, SSU, ITS and *rpb2* sequence data. Bootstrap support values for maximum likelihood (the first value) equal to or greater than 60% and Bayesian posterior probabilities (the second value) equal to or greater than 0.95 are placed near the branches as ML BS/BI PP. The asterisk “*” represents bootstrap support values with 100% ML BS and 1.00 BI PP. The tree is rooted to *Cheilymenia stercorea* AFTOL 148. The ex-type cultures are indicated using “^T^” after strain numbers and the new species introduced in this study are indicated in **bold**.

In the second multi-locus phylogenetic analysis (Figure 2), a total of seven subclasses (Diaporthomycetidae, Hypocreomycetidae, Lulworthiomycetidae, Pisorisporiomycetidae, Savoryellomycetidae, Sordariomycetidae and Xylariomycetidae) in Sordariomycetes, as well as *Pseudocoleodictyospora* and its relatives in Dothideomycetes were included in the dataset, representing 55 isolates and one outgroup taxon (*Cheilymenia stercorea* AFTOL 148). The matrix had 2068 distinct alignment patterns, with 45.12% of completely undetermined characters and gaps. In the RAxML tree, *Coleodictyospora* was phylogenetically distant from *Pseudocoleodictyospora*, although their morphology was quite similar [29]. The relationships of *Obliquifusoideum* were weak with four orders in Savoryellomycetidae (Figure 1), but it was shown to be a genus in Savoryellomycetidae with strong bootstrap support.

In order to assess the phylogenetic position of *Obliquifusoideum* in Savoryellomycetidae, we constructed individual LSU and ITS phylogenetic trees (shown as Supplementary Figures S1 and S2) to enable topological comparison with those derived from the multi-locus datasets. The matrix of the LSU sequence comprised 92 isolates and had 537 distinct alignment patterns, with 14% of undetermined characters or gaps. The matrix of ITS sequence comprised 70 isolates and had 615 distinct alignment patterns, with 30.9% of undetermined characters or gaps. The phylogenetic position of *Obliquifusoideum* was different in all RAxML trees: it clustered with Savoryellales in individual LSU tree (Supplementary Figure S1), clustered with Savoryellales and *Pleurothecium* species of Pleurotheciales in individual ITS tree (Supplementary Figure S2), and clustered with Pleurotheciales in the multi-locus phylogenetic tree (Figure 1); but without bootstrap support in all trees.

### 3.2. Taxonomy of Fungi Colonising Decaying Submerged Wood

#### 3.2.1. Novel Taxa in Pleurotheciaceae

In this section, one new genus and two new species are introduced in Pleurotheciaceae. These taxa are described alphabetically below.

**Sordariomycetes** O.E. Erikss. and Winka, Myconet 1(1): 10 (1997)

**Savoryellomycetidae** Hongsanan, K.D. Hyde and Maharachch., Fungal Diversity 84: 35 (2017)

**Pleurotheciales** Réblová and Seifert, in Réblová, Seifert, Fournier and Štěpánek, Persoonia 37: 63 (2016)

**Pleurotheciaceae** Réblová and Seifert, in Réblová, Seifert, Fournier and Štěpánek, Persoonia 37: 63 (2016)

***Coleodictyospora*** Charles ex Matsush., Matsushima Mycological Memoirs 5: 8 (1987)

*Type species*: Coleodictyospora cubensis Charles ex Matsush.

*Notes*: *Coleodictyospora* was introduced by Charles [30] with a single species *C. cubensis*, but it lacked a Latin diagnosis. Matsushima [31] validated this genus and characterized it as having cylindrical, simple, septate and hyaline conidiophores, monoblastic conidiogenous cells, and transversely oblong or inverse reniform, muriform conidia surrounded by a semi-gelatinous, hyaline sheath. *Berkleasmium micronesicum* was then transferred to *Coleodictyospora* as *C. micronesiaca* based on its very similar morphological traits with *C. cubensis*, but it differs in having smaller conidia (30–42 × 15–18 μm vs. 42–50 × 20–22 μm) and reduced conidiophores [31].

*Coleodictyospora cubensis* was initially collected from North America [30] and subsequently recorded in Brunei [32] and Japan [33]. Nakagiri and Ito [33] named their specimen IFO 32,660 as *C. cubensis* based on the dimensions of conidiophores, conidia and conidial sheaths, as well as the number of conidial septa and the conidiophore attaching point, although it had overlapping conidial size with *C. micronesiaca*. Nakagiri and Ito [33] emphasized that IFO 32,660 might be a novel species considering the thinner conidia (28–48 × 13–19 μm vs. 42–50 × 20–22 μm) and less number of septa (7–11 vs. 8–14) than the type specimen of *C. cubensis*. However, this hypothesis could not be tested without the re-examination of the specimen IFO 32,660 or the molecular data from similar specimens in the same locality (Ishigaki Island, Japan).

*Coleodictyospora micronesiaca* is likely to be a cosmopolitan species as it was recorded in several countries worldwide, including China (Hong Kong, Taiwan) [31,34], Cuba [35], USA (Florida) [36], Mauritius [37], Mexico [38], Micronésia [39], Peru [40], Philippines [37] and Thailand [41]. However, these records were diagnosed solely based on the morphology and lack of support from molecular data, and the descriptions were omitted or briefly noted.

In this study, we isolated a *Coleodictyospora* species from decaying wood submerged in freshwater and provide sequence data for it. Since *C. cubensis* and *C. micronesiaca* lack sequence data in GenBank, we identified our new collection as a novel species in *Coleodictyospora* based on the comparison of their morphology.

**Figure 3:**
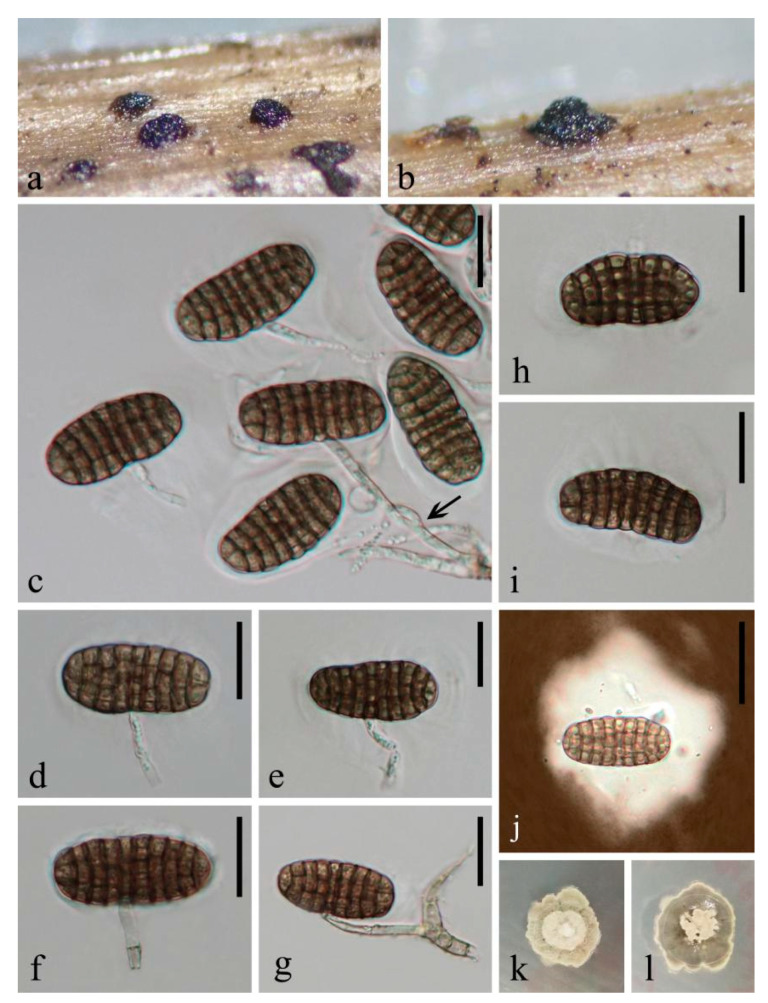
*Coleodictyospora muriformis* (MFLU 18-1544, holotype). (**a**,**b**) sporodochia with a mass of conidia on natural substrate; (**c**) conidia, conidiogenous cells and conidiophores (arrow shows branched conidiophore); (**d**,**e**) conidia with conidiogenous cells; (**f**,**g**) conidiophore bearing conidia; (**h**,**i**) reniform conidia with semi-gelatinous sheaths (h clearly shows the dark brown bands at the conidial transverse septa); (**j**) conidium in Indian Ink showing an irregular sheath; (**k**,**l**) colony on PDA (left-front, right-reverse). Scale bars, (**c**–**i**) 20 μm; (**j**) 30 μm.

**Figure 4:**
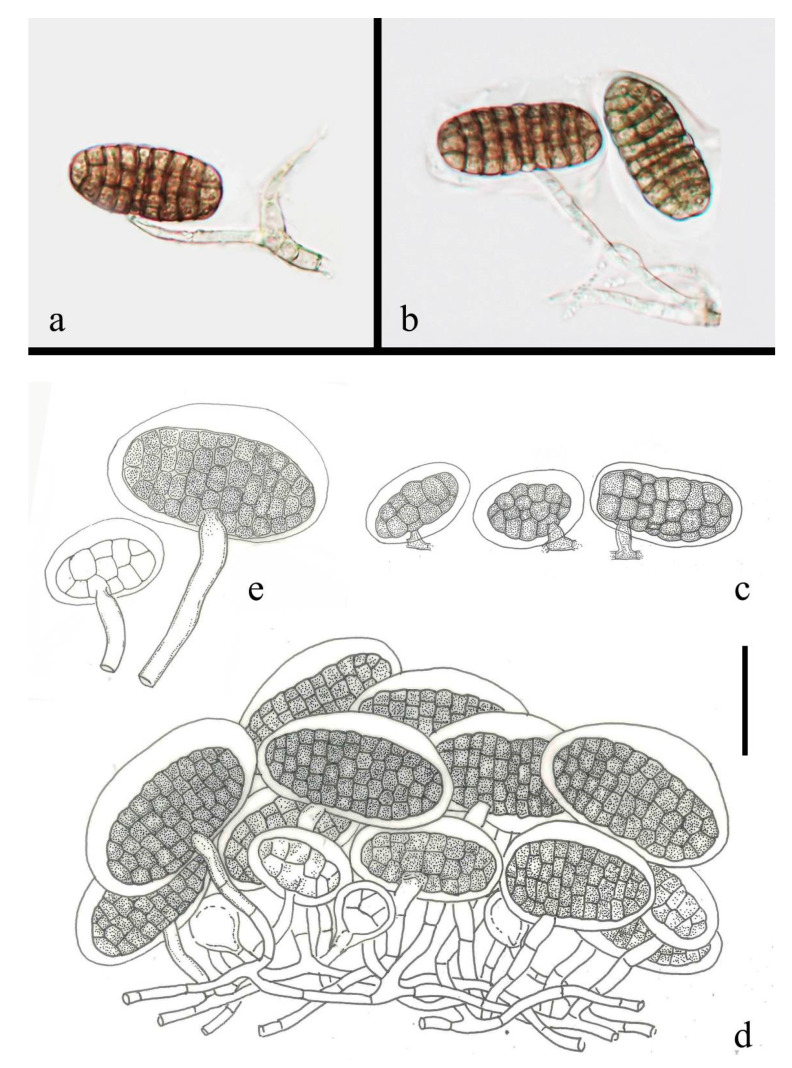
*Coleodictyospora* spp. (**a**,**b** from MFLU 18-1544, holotype. **c**–**e** redrawn from Matsushima [31] and Seifert et al. [42]). (**a**,**b**) *C*. *muriformis* (conidia with branched conidiophores); (**c**) *C. micronesica* (conidia growing on a short subulate conidiogenous cell which directly ascends from the basal mat of sporodochia); (**d**,**e**) *C. cubensis* (d sporodochia bearing a mass of conidia. e conidia with a long conidiogenous cell). Scale bars, (**a**–**e**) 30 μm.

***Coleodictyospora muriformis*** W. Dong, Doilom and K.D. Hyde sp. nov. (Figure 3 and Figure 4a,b)

*Index Fungorum number*: IF558195; Facesoffungi number: FoF 09872

*Etymology*: in reference to the muriform conidia of the fungus

*Holotype*: MFLU 18-1544

*Saprobic* on decaying wood submerged in freshwater. Sexual morph: undetermined. Asexual morph: hyphomycetous. *Colonies* on natural substrate, effuse, gregarious, punctiform, sporodochial, raised, black. *Mycelium* partly immersed in natural substrate, consisting of branched, septate, thin-walled, smooth, pale brown to brown hyphae. *Conidiophores* up to 55 μm long, 3 μm wide, micronematous, mononematous, ascending from the basal mat of sporodochia, subcylindrical, branched, septate, hyaline to pale brown, smooth, thin-walled. *Conidiogenous cells* 7.5–20 × 2.5–3.5 μm ($\bar{x}$ = 13.5 × 3 μm, n = 10), holoblastic, monoblastic, integrated, determinate, terminal, cylindrical, hyaline, smooth, thin-walled. *Conidia* 32–44 × 15.5–19 μm ($\bar{x}$ = 38.5 × 17 μm, n = 100), solitary, acrogenous, generally produced in the middle position, occasionally laterally on conidiophores, and perpendicular to the conidiophores, mostly cylindro-ellipsoidal, sometimes reniform, muriform, dictyoseptate, with (7–)8–9 transverse and (2–)3 longitudinal septa, deeply constricted and with dark brown bands at the transverse septa, slightly constricted and brown to dark brown at the longitudinal septa, often distinctly constricted at the middle where conidiophore attaches to form reniform, brown, smooth, thin-walled, with a hyaline, semi-gelatinous sheath. *Sheaths* well-defined, ellipsoidal, thin at the beginning, 2 μm thick; becoming irregular-shaped, uneven, larger after being mounted in the water, up to 55 μm thick in Indian Ink.

*Culture characteristics*: on PDA, colony irregular, reaching 15 mm diam. in 25 days at room temperature (25–30 °C), surface rough, with dense mycelia, velvety, dry, umbonate in the middle from the side view, edge undulate; from above, dark gray at the margin, pale gray at the middle; from below, dark brown to black at the margin, pale gray at the middle; not producing pigmentation in culture. 

*Material examined*: THAILAND, Satun Province, Khuan Kalong District, Thung Nui Sub-District (6°55′19″ N 100°08′17″ E), on decaying wood submerged in Chang stream originated from Panan Waterfall, 10 May 2018, W. Dong, hat284 (MFLU 18-1544, holotype), ex-type living culture MFLUCC 18-1243 = MFLUCC 18-1279; *ibid*., HKAS 105018, isotype, ex-isotype living culture KUMCC 19-0034 = KUMCC 19-0052.

*Habitat and distribution*: stream is located in tropical rainforest in Southern Thailand with hot and humid climate conditions, shallow and clear, flowing slowly from the Panan Waterfall, surrounded by angiosperms.

*Notes*: *Coleodictyospora muriformis* belongs in *Coleodictyospora* based on the punctiform, sporodochial colonies on the natural substrate, monoblastic conidiogenous cells, and cylindro-ellipsoidal, muriform conidia produced perpendicularly to the conidiophores and with a hyaline, semi-gelatinous sheath [30,31]. *Coleodictyospora muriformis* is easily distinguished from the type species *C. cubensis* by its shorter conidiophores (up to 55 μm long vs. 70–85 μm long), smaller conidia (32–44 × 15.5–19 μm vs. 42–50 × 20–22 μm) and fewer conidial transverse septa ((7–)8–9 vs. 8–14). The transverse septa of the conidia of *C. muriformis* are filled with dark brown bands, while they were neither described nor illustrated in *C. cubensis* [30,31,33,42]. *Coleodictyospora muriformis* has overlapping conidial dimensions with *C. micronesiaca*, but it differs in having long, branched conidiophores (up to 55 μm long) and longer conidiogenous cells (7.5–20 × 2.5–3.5 μm), and the conidiophores attach to the middle of the conidia. In contrast, *C. micronesiaca* lacks conidiophores and the conidiogenous cells are shorter (2–8 × 3–4 μm), which directly ascend from the basal mat of sporodochia; the conidiogenous cells often attach to the end of the conidia. In addition, the conidiogenous cells in *C. micronesiaca* are mostly short subulate, while they are long cylindrical in *C. muriformis*. We therefore introduce *C. muriformis* as new to the genus. A morphological comparison of *Coleodictyospora* species is summarized in Table 2 and a combined figure plate of three species is illustrated in Figure 4.

***Saprodesmium*** W. Dong and Doilom gen. nov.

*Index Fungorum number*: IF558196; Facesoffungi number: FoF 09873

*Etymology*: “saprus” = saprobic, referring to the saprobic lifestyle of the fungus; “desmόs” = bond, link, referring to the aggregated conidia in sporodochia

*Saprobic* on decaying wood submerged in freshwater. Sexual morph: undetermined. Asexual morph: hyphomycetous. *Colonies* on natural substrate, effuse, gregarious, punctiform, sporodochial, raised, black. *Mycelium* partly immersed in natural substrate, consisting of branched, septate, thin-walled, smooth, pale brown to brown hyphae. *Conidiophores* micronematous, mononematous, unbranched, vesiculate, septate. *Conidiogenous cells* holoblastic, monoblastic, integrated, determinate. *Conidia* solitary, obovoid to ellipsoidal, clearly muriform, olivaceous when young, becoming quite blackish with age and obscuring the septa, with several subhyaline basal cells, smooth, thin-walled. *Conidial secession* schizolytic.

*Type species*: Saprodesmium dematiosporum W. Dong, Doilom and K.D. Hyde

*Notes*: The BlastN search of NCBI’s GenBank using the LSU sequence shows *Saprodesmium dematiosporum* has the closest hits with several genera in Pleurotheciaceae, i.e., *Rhexoacrodictys erecta* (KUMCC 20-0194, similarity = 96.68%), *Neomonodictys muriformis* (MFLUCC 16-1136, similarity = 94.11%) and *Pleurothecium obovoideum* (CBS 209.95, similarity = 93.73%). The closest hits using SSU sequence are *Rhexoacrodictys erecta* (KUMCC 20-0194, similarity = 99.54%), *Dematipyriforma aquilaria* (3-11-1, similarity = 99.49%) and *Pleurothecium aquaticum* (B-27, similarity = 99.06%). Based on ITS BlastN search, the closest relatives are however *Phaeoisaria* sp. (BAB-4787, similarity = 97.11%) and *Pleurothecium recurvatum* (CBS 138686, similarity = 96.11%). *Saprodesmium dematiosporum* clusters as an independent branch between *Dematipyriforma* and *Rhexoacrodictys* with high bootstrap support in concatenated LSU-SSU-ITS-*rpb2* phylogeny (100% ML BS/1.00 BI PP, Figure 1) and individual ITS phylogeny (100% ML BS/1.00 BI PP, Supplementary Figure S2). *Saprodesmium dematiosporum* clusters with *Rhexoacrodictys* species in individual LSU phylogeny, but no bootstrap support (Supplementary Figure S1).

*Dematipyriforma* is an endophytic genus comprising a single species *D. aquilaria* [43]. *Dematipyriforma* shares similar morphological characteristics with *Saprodesmium* in having micronematous conidiophores, holoblastic conidiogenous cells and septate conidia. However, they are entirely different genera in the following aspects. The conidiophores of *Dematipyriforma* are hypha-like [43], while they are vesiculate in *Saprodesmium* which are also unique in the family Pleurotheciaceae. The conidia of *Dematipyriforma* are elongate pyriform, 4–5 transverse septate, sometimes 1–2 longitudinal septate, pale grey olivaceous to pale brown, and has rhexolytic conidial secession [43]. In contrast, the conidia of *Saprodesmium* are obovoid to ellipsoidal, irregularly muriform and olivaceous when young, becoming quite blackish with age and obscuring the septa, with several subhyaline basal cells, and has schizolytic conidial secession. In addition, *Saprodesmium* species is saprobe, while *Dematipyriforma* species is endophyte [43].

*Rhexoacrodictys*, typified by *R. erecta*, was introduced for several hyphomycetes characterized by macronematous, long cylindrical conidiophores with percurrent proliferating, monoblastic, integrated, terminal conidiogenous cells, and obovoid, oval or subspherical, muriform, brown to dark brown conidia often with a paler basal cell bearing a small marginal frill derived from the upper portion of the conidiophores and with rhexolytic conidial secession [44]. *Saprodesmium* shares some morphological traits with *Rhexoacrodictys* especially with regards to its muriform and obovoid conidia. *Saprodesmium*, however, has olivaceous conidia and when mature it has quite a blackish pigmentation obscuring the conidial septa. *Rhexoacrodictys* is featured by rhexolytic conidial secession with conidia that have a conspicuous paler basal cell bearing a small marginal frill, while the conidia of *Saprodesmium* secede schizolytically and it instead has several subhyaline, depressed subglobose cells at the base. *Rhexoacrodictys* has macronematous, long cylindrical conidiophores with percurrent proliferating [44,45], whereas *Saprodesmium* has micronematous, short, vesiculate, determinate conidiophores.

Based on the multi-locus and individual phylogenetic analyses, as well as the morphological comparison with the similar taxa in the family, we introduce *Saprodesmium* as a novel genus in Pleurotheciaceae.

**Figure 5:**
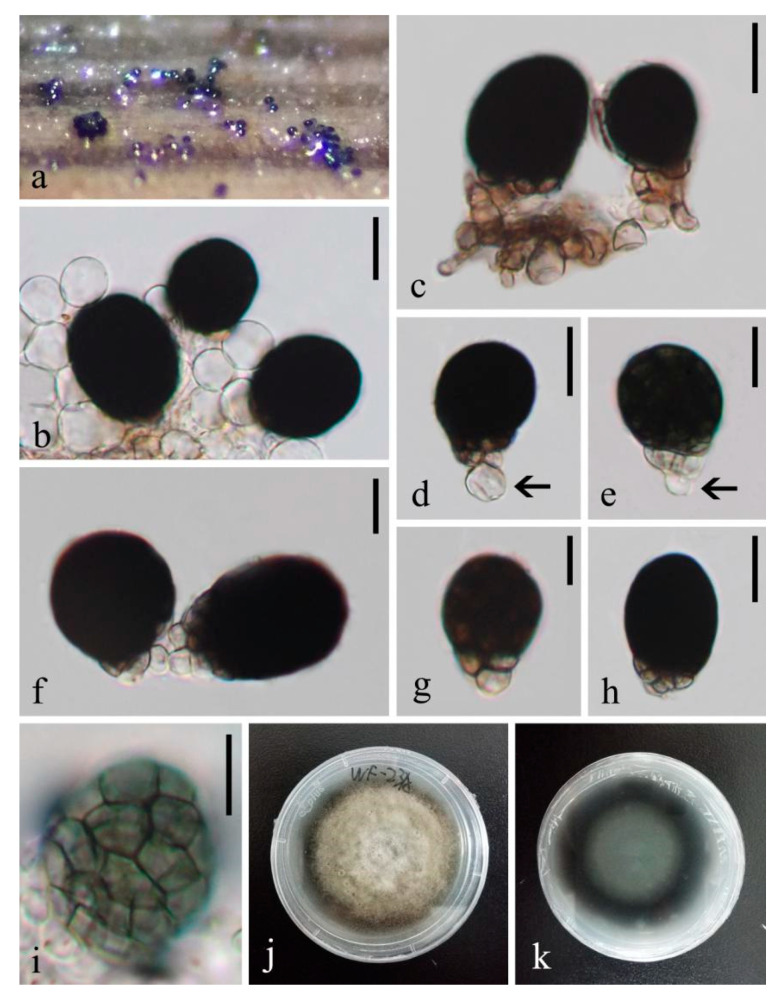
*Saprodesmium dematiosporum* (HKAS 101710, holotype). (**a**) sporodochia with a mass of conidia and scattered conidia on natural substrate; (**b**) conidia and conidiophores; (**c**) conidia attach on pseudoparenchyma of sporodochia; (**d**,**e**) conidia with conidiogenous cells (arrows); (**i**) conidial surface showing muriform patttern; (**j**,**k**) colony on PDA (left-front, right-reverse). Scale bars, (**b**–**e**,**h**) 15 μm; (**f**,**g**,**i**) 10 μm.

***Saprodesmium dematiosporum*** W. Dong, Doilom and K.D. Hyde sp. nov. (Figure 5)

*Index Fungorum number*: IF558197; Facesoffungi number: FoF 09874

*Etymology*: in reference to the dematiaceous conidia

*Holotype*: HKAS 101710

*Saprobic* on decaying wood submerged in freshwater. Sexual morph: undetermined. Asexual morph: hyphomycetous. *Colonies* on natural substrate, effuse, gregarious, punctiform, sporodochial, raised, black. *Mycelium* partly immersed in natural substrate, consisting of branched, septate, thin-walled, smooth, pale brown to brown hyphae. *Conidiophores* micronematous, mononematous, vesiculate, consisted of 1–4 subglobose, smooth, hyaline cells (each cell 8.5–12 μm diam.), unbranched, septate, constricted at the septa, smooth, thin-walled. *Conidiogenous cells* 8–11 μm diam. ($\bar{x}$ = 9.7 μm, n = 10), holoblastic, monoblastic, integrated, determinate, terminal, subglobose, hyaline, smooth, thin-walled. *Conidia* 21–36 × 14.5–27 μm ($\bar{x}$ = 27.5 × 21.5 μm, n = 70), solitary, acrogenous, obovoid to ellipsoidal, subglobose, clearly muriform, olivaceous when young, becoming quite blackish with age and obscuring the septa, with several subhyaline to pale brown basal cells, smooth, thin-walled. *Conidial secession* schizolytic.

*Culture characteristics*: on PDA, colony circular, reaching 50 mm diam. in 30 days at room temperature (25–30 °C), surface rough, with dense mycelia, dry, raised from the side view, edge entire; from above, dark gray at the margin, pale gray to white at the middle; from below, black at the margin, dark olivaceous at the middle; not producing pigmentation in culture.

*Material examined*: CHINA, Yunnan Province, Pingbian District (22°59′13″ N 103°40′30″ E), on decaying wood submerged in an unnamed stream originated from Dawei Mountain Nature Reserve, 20 September 2017, W. Dong, WF23A (HKAS 101710, holotype), ex-type living culture KUMCC 18-0059; *ibid*., MFLU 18-1165, isotype.

*Habitat and distribution*: stream is nearby Nature Reserve in Southern Yunnan of Yunnan-Kweichow Plateau, shallow and clear, flowing rapidly from the Dawei Mountain, surrounded by angiosperms.

#### 3.2.2. Novel Taxa in Savoryellomycetidae

In this section, one new genus with one new species are introduced and phylogenetically referred to Savoryellomycetidae genera *incertae sedis*.

***Obliquifusoideum*** W. Dong, Doilom and K.D. Hyde gen. nov.

*Index Fungorum number*: IF558198; Facesoffungi number: FoF 09875

*Etymology*: in reference to its neck growing oblique to the host substrate and fusoid ascospores

*Saprobic* on decaying wood submerged in freshwater. Sexual morph: *Ascomata* superficial, ellipsoidal, black, coriaceous, ostiolate, with a lateral neck. *Necks* hyaline to dark, subcylindrical, oblique or horizontal to the host substrate. *Peridium* thin, soft, comprising several layers of brown, thin-walled cells of *textura angularis*. *Paraphyses* tapering towards the apex, dense, hypha-like, septate, unbranched, hyaline. *Asci* 8-spored, unitunicate, cylindrical, short pedicellate, with a small, refractive, barrel- or jar-shaped, apical ring, persistent. *Ascospores* uniseriate, fusoid, septate, hyaline, thin-walled. Asexual morph: undetermined.

*Type species*: Obliquifusoideum guttulatum W. Dong, Doilom and K.D. Hyde

*Notes*: The BlastN search of NCBIs GenBank using LSU sequence shows *Obliquifusoideum guttulatum* has the closest hits with several genera in Pleurotheciaceae, but with low percentage similarity, i.e., *Melanotrigonum ovale* (CBS 138743, similarity = 92.06%), *Pleurotheciella saprophytica* (MFLUCC 16-1251, similarity = 92.03%), *Phaeoisaria annesophieae* (MFLU 19-0531, similarity = 91.86%) and *Sterigmatobotrys rudis* (DAOM 229838, similarity = 91.83%). The closest hits using SSU sequence are several genera in Pleurotheciaceae, i.e., *Dematipyriforma aquilaria* (3-11-1, similarity = 98.13%), *Phaeoisaria clematidis* (MFLUCC 18-1017, similarity = 97.99%) and *P. fasciculata* (DAOM 230055, similarity = 97.92%). The closest hits using ITS sequence are however several genera in Conioscyphales and Pleurotheciales, i.e., *Pleurothecium recurvatum* (DAOM 230069, similarity = 98.75%), *Conioscypha varia* (CBS 604.70, similarity = 94.89%) and *Neomonodictys muriformis* (MFLUCC 16-1136, similarity = 93.19%).

The placement of *Obliquifusoideum guttulatum* is different in multi-locus and individual LSU and ITS phylogenetic trees and lacks significant support in all trees. *Obliquifusoideum guttulatum* is revealed as a sister taxon of Pleurotheciales in the multi-locus analysis of concatenated LSU-SSU-ITS-*rpb2* matrix (Figure 1), while it clusters with Savoryellales in the individual LSU phylogeny (Supplementary Figure S1); Savoryellales and *Pleurothecium* species in the individual ITS phylogeny (Supplementary Figure S2). *Obliquifusoideum* is similar to *Helicoascotaiwania* in Pleurotheciales, and *Ascotaiwania*, *Neoascotaiwania* and *Savoryella* in Savoryellales. They generally have dark ascomata with a lateral neck, which is oblique or horizontal to the host substrate, and septate ascospores. However, the ascospores of the four genera are mostly ellipsoidal and versicolorous with dark middle cells and hyaline polar cells [2,3,4,46,47]. In contrast, *Obliquifusoideum* has fusoid and evenly hyaline ascospores. The morphological differences and the independent lineage in the multi-locus and individual phylogenetic trees therefore support *Obliquifusoideum* as a new genus.

Although the relationships of *Obliquifusoideum* were weak with four orders in Savoryellomycetidae (Figure 1), it was shown to be a genus in Savoryellomycetidae with strong bootstrap support (Figure 2). We consider it is wise to refer *Obliquifusoideum* to Savoryellomycetidae genera *incertae sedis* for now, until its phylogeny is better resolved with additional taxon sampling followed by divergence time estimates studies.

**Figure 6:**
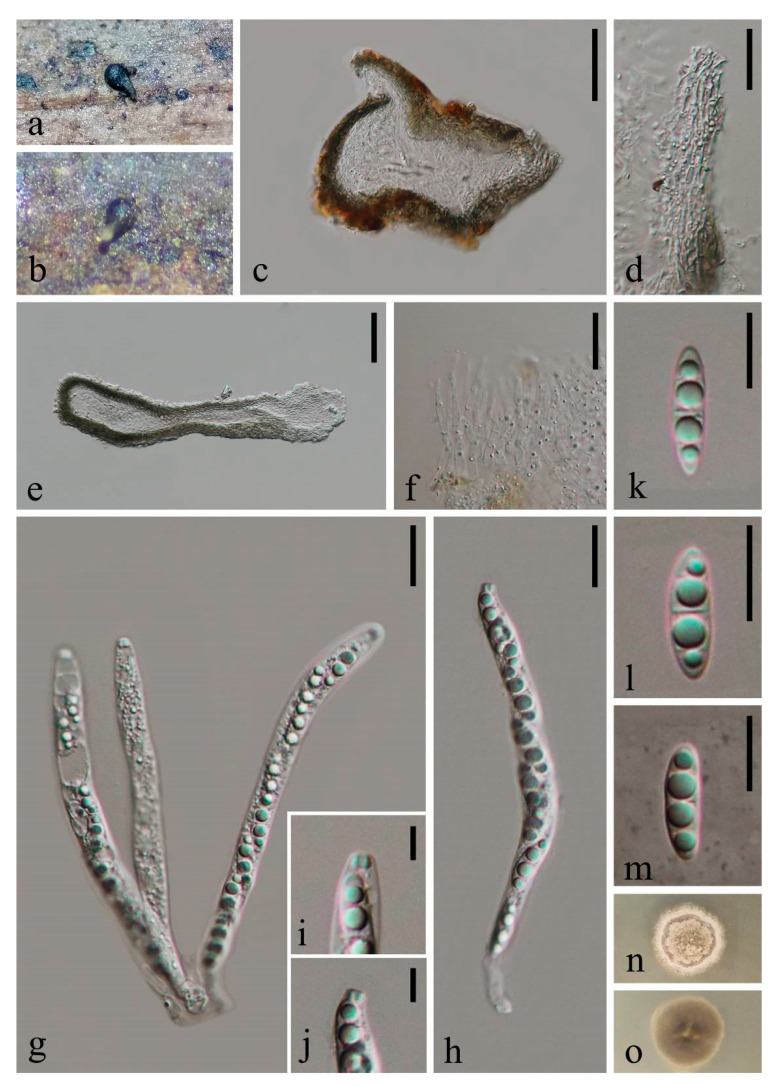
*Obliquifusoideum guttulatum* (MFLU 18-1575, holotype). (**a**,**b**) ascomata lying on submerged wood; (**c**) section of ascoma; (**d**) peridium; (**e**) section of neck; (**f**) paraphyses; (**g**,**h**) unitunicate asci; (**i**,**j**) apical rings; (**k**–**m**) ascospoers (m ascospore in Indian Ink); (**n**,**o**) colony on PDA (up-front, down-reverse). Scale bars, (**c**) 50 μm; (**d**,**f**) 20 μm; (**e**) 30 μm; (**g**,**h**) 15 μm; (**i**,**j**) 5 μm; (**k**–**m**) 10 μm.

***Obliquifusoideum guttulatum*** W. Dong, Doilom and K.D. Hyde sp. nov. (Figure 6)

*Index Fungorum number*: IF558199; Facesoffungi number: FoF 09876

*Etymology*: in reference to the guttulate ascospores of the fungus

*Holotype*: MFLU 18-1575

*Saprobic* on decaying wood submerged in freshwater. Sexual morph: *Ascomata* 100–120 μm high, 155–170 μm diam., scattered, superficial, ellipsoidal, black, coriaceous, ostiolate, with a lateral neck, ejecting asci and ascospores soon during incubation and becoming empty. *Necks* 160–180 μm long, 17–30 μm wide, hyaline to black, subcylindrical, oblique or horizontal to the host substrate. *Peridium* thin, 8–20 μm thick, soft, comprising several layers of pale brown, thin-walled cells of *textura angularis*, dark brown outwards. *Paraphyses* 3.5–5 μm wide, tapering towards the apex, dense, hypha-like, septate, unbranched, hyaline, embedded in a gelatinous matrix. *Asci* 97–110 × 7.3–7.7 μm ($\bar{x}$ = 105 × 7.5 μm, n = 10), 8-spored, unitunicate, cylindrical, slightly narrower and truncate at the apex, short pedicellate, with a small, distinct, refractive, barrel- or jar-shaped, apical ring, 2 × 2.7 μm, persistent. *Ascospores* 14–17.5 × 4.3–5 μm ($\bar{x}$ = 15.5 × 4.6 μm, n = 10), overlapping uniseriate, fusoid, straight or slightly curved, one median septate, with two additional obscure septate at two sides, guttulate, hyaline, thin and smooth-walled, without a gelatinous sheath. Asexual morph: undetermined.

*Culture characteristics*: on PDA, colony circular, reaching 8 mm diam. in 48 days at room temperature (25–30 °C), surface rough, with dense mycelia, dry, rigid, umbonate from the side view, edge entire; from above, creamy at the margin, dark grey to dark brown at the middle, brown at the center; dark brown from below; not producing pigmentation in culture.

*Material examined*: THAILAND, Songkhla Province, Rattaphum District, Khao Phra Sub-District (7°00′03″ N 100°08′33″ E), on decaying wood submerged in a stream originated from Borriphat Waterfall, 10 May 2018, W. Dong, hat138 (MFLU 18-1575, holotype), ex-type living culture MFLUCC 18-1233; *ibid*., HKAS 105007, isotype, ex-isotype living culture KUMCC 19-0023.

*Habitat and distribution*: stream is located in tropical rainforest in Southern Thailand with hot and humid climate conditions, shallow and clear, flowing slowly from the Borriphat Waterfall, surrounded by angiosperms.

## 4. Discussion

Doilom et al. [29] established a novel genus *Pseudocoleodictyospora* to accommodate three hyphomycetous species collected from the bark of living *Tectona grandis* (teak) and distinguished them from *Coleodictyospora* by the absence of a hyaline sheath. This establishment, however, lacks the support from the DNA sequence data of *Coleodictyospora*. The presence of conidial sheath as a criterion for delimiting two genera is interesting as this is often used for species delimitation in classification, such as species in *Astrosphaeriella*, *Dictyosporium*, *Kirschsteiniothelia* and *Natipusilla* [48]. In this study, we collected a freshwater hyphomycetous species which has very similar morphs to *Pseudocoleodictyospora*, but is characterized by a hyaline sheath. This peculiar phenotype further confirms it as a novel species in *Coleodictyospora*, namely *C. muriformis* (see notes of *C. muriformis*). On the basis of DNA-based phylogeny, *Coleodictyospora muriformis* is phylogenetically distant from *Pseudocoleodictyospora* (Pseudocoleodictyosporaceae, Pleosporales) and clusters as a member of the Pleurotheciales (Figure 2). This study further confirms the Doilom et al. [29] taxonomic assumption of establishing a novel genus based on the presence of conidial sheath with the support from the DNA sequence data. Amazingly, *Coleodictyospora* and *Pseudocoleodictyospora* are positioned in two different classes Sordariomycetes and Dothideomycetes, respectively (Figure 2), though they are quite similar except in terms of the conidial sheath. Nevertheless, it is not advisable to use conidial sheath as a criterion segregating species at a higher taxonomic level as it is often an unstable characteristic, especially among freshwater species such as *Caryospora submersa* and *Pseudoastrosphaeriella bambusae* [48].

In our multi-locus phylogenetic tree (Figure 1), *Coleodictyospora* is affiliated to *Neomonodictys muriformis* and *Pleurothecium obovoideum*. *Coleodictyospora* is similar to *Neomonodictys* in having muriform conidia, but they are entirely different genera. The conidia of *Neomonodictys* are subglobose to globose, comprising several subglobose cells, which are irregularly arranged in the conidia, pale brown when immature, producing black pigmentation and obscuring the conidial septa, with a protruding basal cell which attaches to the conidiophore [8]. In contrast, *Coleodictyospora* has cylindro-ellipsoidal conidia, with (7–)8–9 transverse and (2–)3 longitudinal septa, deeply constricted and with dark brown bands at the transverse septa, generally produced in the middle position and are perpendicular on the conidiophore. *Pleurothecium obovoideum* was proposed based on a known species, *Ramichloridium obovoideum* [49]. In the phylogenetic tree of Arzanlou et al. [49], they showed that the strain CBS 209.95 of *R. obovoideum* clustered with the sexual morph of *Pleurothecium recurvatum* (type species) and its morphological characteristics fit well with *Pleurothecium* and *R. obovoideum* was therefore transferred to *Pleurothecium*, namely *P. obovoideum*. However, with more species in *Pleurothecium*, *P. obovoideum* was reported to be distant from *P. recurvatum* and clustered with *Neomonodictys* in a well-supported clade [6,8]. The re-assessment of *P. obovoideum* is pending, however, its pleurothecium-like morphological characteristics [49] warrant it cannot be congeneric with *Coleodictyospora*.

Interestingly, we found that the conidia of *Coleodictyospora* are quite similar to the ascospores of a sexual species *Boerlagiomyces websteri*. *Boerlagiomyces* were recognized in Tubeufiaceae [11,50,51] and confirmed with DNA sequence data derived from a reference specimen of *Boerlagiomyces macrospora* [29]. However, *B. websteri* represented by a putative strain BCC 3834 clustered with several apothecial taxa in Pezizomycotina [52], and Boonmee et al. [50] had some doubts on this species because of its perithecial characteristic. Therefore, the accurate phylogenetic position of *B. websteri* is still questionable. Although the soft, membranous, setose ascomata and large dictyosporous ascospores of *B. websteri* fit with the features of *Boerlagiomyces* [50], the two-spored asci are unusual compared with the eight-spored asci of the type species *B. velutinus* [50]. Whether *B. websteri* has close phylogenetic relationships with *Coleodictyospora* in Savoryellomycetidae is pending and has to be resolved.

On the basis of morphology, we initially considered *Obliquifusoideum* as a member of Annulatascales due to its black ascomata with a lateral neck which is oblique or horizontal to the host substrate, hypha-like paraphyses with tapering apex, cylindrical asci with a distinct, refractive apical ring, and fusoid, hyaline ascospores. It is of interest, however, that *Obliquifusoideum* clusters in Savoryellomycetidae with relationships to Pleurotheciales and Savoryellales, which is distant from Annulatascales and annulatascales-like taxa in Diaporthomycetidae (Figure 2). It is not unexpected that *Obliquifusoideum* with annulatascales-like morphology can be discovered in another subclass Savoryellomycetidae as Annulatascales is commonly recognized to be polyphyletic and the species are often encountered from freshwater habitats [53,54]. It is reasonable that *Obliquifusoideum* is placed in Savoryellomycetidae due to its dark ascomata with an oblique or horizontal neck, and phragmoseptate conidia which are the sexual features of the other two members Pleurotheciales and Savoryellales.

DNA-based phylogeny has helped to provide better insights into the taxonomy of Pleurotheciales and a recommendation of species boundaries was established [55], leading to recent classification updates. *Rhexoacrodictys*, which was treated as a genus in Savoryellales by Xia et al. [56], was accepted in Pleurotheciales in a later phylogenetic study [6]. Our phylogenetic results corroborate those of Luo et al. [6], and our new genus *Saprodesmium* forms a well-supported lineage basal to *Rhexoacrodictys* in Pleurotheciales (Figure 1). The BlastN search of *Saprodesmium* using SSU sequence in NCBIs GenBank reveals a high similarity (99.49%) with a hyphomycetous species *Dematipyriforma aquilaria*. *Dematipyriforma*, typified by *D. aquilaria*, was isolated as an endophyte from the trunk of *Aquilaria crassna*, producing dark muriform conidia [43]. *Dematipyriforma* was placed in Savoryellales in the phylogenetic tree of Sun et al. [43], however, they did not include other related orders (Conioscyphales, Fuscosporellales and Pleurotheciales) in Savoryellomycetidae. Our multi-locus phylogeny places *Dematipyriforma* as a sister genus to *Rhexoacrodictys* and *Saprodesmium* with good bootstrap support in Pleurotheciales (99% ML BS/1.00 BI PP, Figure 1). On the other hand, the muriform conidia of *Dematipyriforma* are similar to *Neomonodictys* in Pleurotheciales. According to this morphological trait and phylogenetic result, we accept *Dematipyriforma* in Pleurotheciales. Besides saprobes and few opportunistic human pathogens, *Dematipyriforma* is the only presently known endophytic genus in the order, which increases our understanding of different life modes of Pleurotheciales.

Freshwater fungi are a unique group of organisms with a special ability to survive and grow on submerged wood in water by producing soft rot cavities [57,58]. There is very little overlap between the fungi growing on wood submerged in freshwater and those on adjacent stream sides [59,60]. Thus, we are continually finding novel taxa from this unique habitat and since streams are often disparate elements, we are likely to discover many more which will improve our understanding of fungal classification [61]. Freshwater appears to be an ecologically important niche for species in Pleurotheciales [62]. Almost all *Pleurotheciella* species were collected from freshwater [3,4,6,9,10,13], as well as some species from *Helicoascotaiwania*, *Phaeoisaria*, *Pleurothecium* and *Sterigmatobotrys* [6,10,12,15,63].

## Figures and Tables

**Table 1:** Taxa used in the phylogenetic analyses and their corresponding GenBank accession numbers.

Taxon	Voucher/Culture	GenBank Accession Numbers			
		LSU	SSU	ITS	*rpb2*
*Achroceratosphaeria potamia*	CBS 125414	GQ996538	GQ996541	MH863679	KM588908
*Adelosphaeria catenata*	CBS 138679^T^	KT278707	KT278692	KT278721	KT278743
*Anapleurothecium botulisporum*	FMR 11490^T^	KY853483	-	KY853423	-
*Arecophila bambusae*	HKUCC 4794	AF452038	AY083802	-	-
*Ascotaiwania latericolla*	ICMP 22739^T^	MN699407	-	MN699390	MN704312
*Ascotaiwania lignicola*	NIL 00005	HQ446364	HQ446284	-	HQ446419
*Bactrodesmiastrum obovatum*	FMR 6482	FR870266	-	-	-
*Bactrodesmiastrum pyriforme*	FMR 11931	HE646637	-	-	-
*Bactrodesmium diversum*	CBS 144080	MN699415	MN699371	MN699355	MN704294
*Bactrodesmium obovatum*	CBS 144078	MN699425	MN699376	MN699396	-
*Bactrodesmium pallidum*	CBS 145349	MN699429	MN699380	MN699364	MN704302
*Brachysporiella setosa*	HKUCC 3713	AF132334	-	-	-
*Canalisporium exiguum*	SS 00809	GQ390281	GQ390266	-	HQ446436
*Canalisporium grenadoideum*	BCC 20507	GQ390267	GQ390252	-	HQ446420
*Cercophora caudata*	CBS 606.72	AY999113	DQ368659	AY999135	DQ368646
*Cercophora newfieldiana*	SMH 3303	AY780062	-	-	AY780167
*Cercophora thailandica*	MFLUCC 12-0845	KU863127	KU872131	-	KU940176
*Cheilymenia stercorea*	AFTOL 148	AY544661	AY544705	-	-
***Coleodictyospora muriformis***	**MFLUCC 18-1243^T^**	**MW981648**	**MW981704**	**MW981642**	**-**
***Coleodictyospora muriformis***	**MFLUCC 18-1279^T^**	**MW981649**	**MW981705**	**MW981643**	**-**
*Conioscypha japonica*	CBS 387.84	AY484514	JQ437438	-	-
*Conioscypha lignicola*	CBS 335.93^T^	AY484513	JQ437439	-	JQ429260
*Conioscypha peruviana*	ILL 41202^T^	KF781539	-	-	-
*Conioscypha varia*	CBS 604.70	MH871656	-	MH859869	-
*Cosmospora arxii*	CBS 748.69	MH871181	-	NR-145062	HQ897725
*Dematiosporium aquaticum*	MFLU 18-1641^T^	MK835855	-	-	MN194029
*Dematipyriforma aquilaria*	CGMCC 3.17268^T^	KJ138623	KJ138622	KJ138621	-
*Diaporthe cyatheae*	YMJ 1364	JX570891	JX570890	-	JX570893
*Diaporthe eres*	AR 3538	AF408350	-	-	-
*Diaporthe xishuangbanica*	LC6744	KY011862	-	-	-
*Doratomyces stemonitis*	AFTOL-ID 1380	DQ836907	DQ836901	-	-
*Entosordaria perfidiosa*	BW3	MF488992	-	-	MF489002
*Fuscosporella pyriformis*	MFLUCC 16-0570^T^	KX550896	KX550900	-	KX576872
*Fusicolla aquaeductuum*	KUMCC 18-0015	MH087221	-	MH087219	-
*Helicoascotaiwania farinosa*	DAOMC 241947	JQ429230	-	JQ429145	-
*Helicoascotaiwania farinosa*	ILLS 53605^T^	AY094189	-	-	-
*Helicoascotaiwania farinosa*	P2-6	AY316357	-	-	-
*Helicoascotaiwania lacustris*	CBS 145963^T^	MN699430	MN699382	MN699399	MN704304
*Helicoascotaiwania lacustris*	CBS 145964	MN699431	MN699383	MN699400	MN704305
*Helicoascotaiwania lacustris*	CBS 146144	MN699432	MN699384	MN699401	MN704306
*Melanotrigonum ovale*	CBS 138744	KT278710	KT278697	KT278725	KT278746
*Melanotrigonum ovale*	CBS 138815	KT278711	KT278698	KT278722	KT278747
*Melanotrigonum ovale*	CBS 138743^T^	KT278709	KT278696	KT278724	KT278745
*Microascus trigonosporus*	AFTOL-ID 914	DQ470958	DQ471006	DQ491513	-
*Mucispora obscuriseptata*	MFLUCC 15-0618^T^	KX550892	KX550897	-	KX576870
*Nectria nigrescens*	AR 4211	HM484720	JN939647	HM484707	JQ014123
*Neoascotaiwania fusiformis*	MFLUCC 15-0621^T^	KX550893	-	MG388215	KX576871
*Neoascotaiwania limnetica*	CBS 126576	KY853513	KT278689	KY853452	MN704308
*Neoascotaiwania terrestris*	CBS 142291^T^	KY853515	KY853547	KY853454	-
*Neomonodictys muriformis*	MFLUCC 16-1136^T^	MN644485	-	MN644509	-
*Neoroussoella alishanense*	MFLUCC 11-0190	MN028398	-	MN028394	-
*Neoroussoella bambusae*	MFLUCC 11-0124	KJ474839	-	KJ474827	KJ474856
*Neotorula submersa*	KUMCC 15-0280	KX789217	-	KX789214	-
*Neurospora crassa*	MUCL 19026	AF286411	X04971	-	-
***Obliquifusoideum guttulatum***	**MFLUCC 18-1233^T^**	**MW981650**	**MW981706**	**MW981645**	**-**
*Paracremonium binnewijzendii*	CBS 143277	MG250174	-	NR-157491	-
*Parathyridaria percutanea*	CBS 868.95	KF366449	KF366451	KF322118	KF366452
*Phaeoisaria annesophieae*	MFLU 19-0531	MT559084	-	MT559109	-
*Phaeoisaria aquatica*	MFLUCC 16-1298^T^	MF399254	-	MF399237	MF401406
*Phaeoisaria clematidis*	MFLUCC 18-1017	MW132065	MW132063	MW131990	-
*Phaeoisaria fasciculata*	CBS 127885^T^	KT278705	KT278693	KT278719	KT278741
*Phaeoisaria fasciculata*	DAOM 230055	KT278706	KT278694	KT278720	KT278742
*Phaeoisaria pseudoclematidis*	MFLUCC 11-0393^T^	KP744501	KP753962	KP744457	-
*Phaeoisaria sedimenticola*	CGMCC 3.14949^T^	JQ031561	-	JQ074237	-
*Phragmocephala stemphylioides*	DAOM 673211	KT278717	-	KT278730	-
*Pisorisporium cymbiforme*	PRM 924378	KM588902	KM588899	-	KM588905
*Pleurotheciella aquatica*	MFLUCC 17-0464^T^	MF399253	MF399220	MF399236	MF401405
*Pleurotheciella centenaria*	DAOM 229631^T^	JQ429234	JQ429246	JQ429151	JQ429265
*Pleurotheciella erumpens*	CBS 142447^T^	MN699435	MN699387	MN699406	MN704311
*Pleurotheciella fusiformis*	MFLUCC 17-0113^T^	MF399250	MF399218	MF399233	MF401403
*Pleurotheciella guttulata*	KUMCC 15-0442	MF399256	MF399222	MF399239	MF401408
*Pleurotheciella guttulata*	KUMCC 15-0296^T^	MF399257	MF399223	MF399240	MF401409
*Pleurotheciella krabiensis*	MFLUCC 16-0852^T^	MG837013	MG837023	MG837018	-
*Pleurotheciella lunata*	MFLUCC 17-0111^T^	MF399255	MF399221	MF399238	MF401407
*Pleurotheciella rivularia*	CBS 125238^T^	JQ429232	JQ429244	JQ429160	JQ429263
*Pleurotheciella rivularia*	CBS 125237	JQ429233	JQ429245	JQ429161	JQ429264
*Pleurotheciella saprophytica*	MFLUCC 16-1251^T^	MF399258	MF399224	MF399241	MF401410
*Pleurotheciella submersa*	DLUCC 0739	MF399259	MF399225	MF399242	MF401411
*Pleurotheciella submersa*	MFLUCC 17-1709^T^	MF399260	MF399226	MF399243	MF401412
*Pleurotheciella sympodia*	MFLUCC 18-1408	MW981652	-	MW981644	-
*Pleurotheciella sympodia*	MFLUCC 15-0996	MW981651	MW981703	MW981641	-
*Pleurotheciella sympodia*	MFLUCC 18-0658	MT559086	MT559094	MT555418	-
*Pleurotheciella sympodia*	MFLUCC 18-0983	MT555425	MT555734	MT555419	-
*Pleurotheciella sympodia*	KUMCC 19-0213	MT555426	-	MT555420	-
*Pleurotheciella tropica*	MFLUCC 16-0867^T^	MG837015	MG837025	MG837020	-
*Pleurotheciella uniseptata*	DAOM 673210^T^	KT278716	-	KT278729	-
*Pleurotheciella uniseptata*	KUMCC 15-0407	MF399248	-	MF399231	MF401401
*Pleurothecium aquaticum*	MFLUCC 17-1331^T^	MF399263	-	MF399245	-
*Pleurothecium aquaticum*	B-27	MK835854	MK834786	-	-
*Pleurothecium floriforme*	MFLUCC 15-0628	KY697277	KY697279	KY697281	-
*Pleurothecium obovoideum*	CBS 209.95	EU041841	-	EU041784	-
*Pleurothecium pulneyense*	MFLUCC 16-1293	MF399262	MF399228	-	MF401414
*Pleurothecium recurvatum*	DAOM 230069	JQ429238	JQ429252	JQ429157	JQ429269
*Pleurothecium semifecundum*	CBS 131271^T^	JQ429240	JQ429254	JQ429159	JQ429270
*Podosordaria tulasnei*	CBS 128.80	KT281897	-	-	-
*Pseudoascotaiwania persoonii*	A57-14C	AY094190	-	-	-
*Pseudocoleodictyospora sukhothaiensis*	MFLUCC 12-0554	KU764710	KU712471	KU712440	KU712493
*Pseudocoleodictyospora tectonae*	MFLUCC 12-0385	KU764709	KU712461	KU712443	KU712491
*Pseudocoleodictyospora tectonae*	MFLUCC 12-0387	KU764704	KU712462	KU712444	KU712492
*Pseudocoleodictyospora thailandica*	MFLUCC 12-0565	KU764701	KU712472	KU712441	KU712494
*Pseudoneurospora amorphoporcata*	CBS 626.80	FR774287	-	-	-
*Rhexoacrodictys erecta*	HSAUPmyr4622	KX033556	KX033526	KU999964	-
*Rhexoacrodictys erecta*	HSAUPmyr6489	KX033555	KX033525	KU999963	-
*Rhexoacrodictys erecta*	KUMCC 20-0194	MT559123	-	MT555421	-
*Rhexoacrodictys fimicola*	HMAS47737	KX033553	KX033522	KU999960	-
*Rhexoacrodictys fimicola*	HMAS43690	KX033550	KX033519	KU999957	-
*Roussoella nitidula*	MFLUCC 11-0182	KJ474843	-	KJ474835	KJ474859
***Saprodesmium dematiosporum***	**KUMCC 18-0059^T^**	**MW981647**	**MW981707**	**MW981646**	**-**
*Savoryella aquatica*	SS 03801	HQ446372	HQ446290	-	HQ446441
*Savoryella lignicola*	NF00204	HQ446378	HQ446300	HQ446357	-
*Savoryella longispora*	SAT 00322	HQ446380	HQ446302	HQ446359	HQ446450
*Savoryella paucispora*	SAT 00866	HQ446381	HQ446303	-	HQ446451
*Savoryella verrucosa*	SS 00052	HQ446374	-	HQ446353	HQ446445
*Savoryella yunnanensis*	MFLUCC 18-1395^T^	MK411422	MK411423	-	-
*Sordaria fimicola*	CBS 508.50	AY681160	-	-	-
*Sterigmatobotrys macrocarpa*	PRM 915682	GU017317	JQ429255	JQ429153	-
*Sterigmatobotrys macrocarpa*	DAOM 230059	GU017316	-	JQ429154	JQ429271
*Sterigmatobotrys rudis*	DAOM 229838	JQ429241	JQ429256	JQ429152	JQ429272
*Sterigmatobotrys uniseptata*	MFLUCC 15-0358^T^	MK835850	MK834784	MK878379	-
*Subglobosporium tectonae*	MFLUCC 12-0393	KU764703	KU712464	KU712445	KU712485
*Subglobosporium tectonae*	MFLUCC 12-0390	KU764702	KU712463	KU712446	KU712495
*Thyridaria broussonetiae*	CBS 141481	KX650568	-	NR-147658	KX650586
*Torula aquatica*	MFLUCC 16-1115	MG208146	-	MG208167	MG207977
*Torula herbarum*	CPC 24114	KR873288	-	KR873260	-
*Triadelphia uniseptata*	TA06NZ-142	KT278718	-	-	-
*Tubakia seoraksanensis*	CBS 127490	KP260499	-	-	-
*Xylaria hypoxylon*	CBS 122620	KY610495	-	KY610407	KY624231
*Zalerion maritima*	FCUL280207CP1	JN886806	KT347203	KT347216	-
*Zalerion xylestrix*	309156	EU848592	EU848591	-	-

The ex-type cultures are indicated using “^T^” after strain numbers and newly generated sequences are indicated in **bold**.

**Table 2:** Morphological comparison of *Coleodictyospora* species (on natural substrate) (update from Nakagiri and Ito [33]).

	*C. cubensis*	*C. micronesica*	*C. muriformis*
Conidiophores	70–85 × 3.5–5 μm	Reduced, 2–8 × 3–4 μm (on CMA)	Up to 55 μm long, 3 μm wide
Conidiophore attaching point	Middle	End	Middle
Conidia	42–50 × 20–22 μm	30–40 × 13–16 μm	32–44 × 15.5–19 μm
Conidial sheaths	55–60 × 40–45 μm	Present, but not measured	Up to 55 μm thick in Indian Ink
Conidial transverse septa	8–14	6–9	(7–)8–9
Conidial septa with or without dark bands	Not mentioned	Not mentioned	With dark brown bands

## Data Availability

The data generated from this study can be found in the Index Fungorum (http://www.indexfungorum.org/names/names.asp, accessed on 1 August 2021) and GenBank (https://www.ncbi.nlm.nih.gov/nuccore, accessed on 1 August 2021).

## Supplementary Materials

The following are available online at https://www.mdpi.com/article/10.3390/jof7090711/s1, Figure S1: Phylogenetic tree generated from maximum likelihood analysis (RAxML) based on LSU sequence data, Figure S2: Phylogenetic tree generated from maximum likelihood analysis (RAxML) based on ITS sequence data.

## References

[1] Hongsanan S., Maharachchikumbura S., Hyde K.D., Samarakoon M.C., Jeewon R., Zhao Q., Al-Sadi A., Bahkali A.H. (2017). An updated phylogeny of Sordariomycetes based on phylogenetic and molecular clock evidence. Fungal Divers..

[2] Dayarathne M.C., Maharachchikumbura S., Jones E.B.G., Dong W., Devadatha B., Yang J., Ekanayaka A.H., De Silva W., Sarma V.V., Al-Sadi A. (2019). Phylogenetic Revision of Savoryellaceae and Evidence for Its Ranking as a Subclass. Front. Microbiol..

[3] Réblová M., Restrepo M.H., Fournier J., Nekvindová J. (2020). New insights into the systematics of *Bactrodesmium* and its allies and introducing new genera, species and morphological patterns in the Pleurotheciales and Savoryellales (Sordariomycetes). Stud. Mycol..

[4] Réblová M., Seifert K., Fournier J., Štěpánek V. (2016). Newly recognised lineages of perithecial ascomycetes: The new orders Conioscyphales and Pleurotheciales. Pers. Mol. Phylogeny Evol. Fungi.

[5] Guarro J., Vieira L.A., De Freitas D., Gené J., Zaror L., Hofling-Lima A.L., Fischman O., Zorat-Yu C., Figueras M.J. (2000). *Phaeoisaria clematidis* as a Cause of Keratomycosis. J. Clin. Microbiol..

[6] Luo Z.-L., Hyde K.D., Liu J.-K., Maharachchikumbura S., Jeewon R., Bao D.-F., Bhat D.J., Lin C.-G., Li W.-L., Yang J. (2019). Freshwater Sordariomycetes. Fungal Divers..

[7] Hernández-Restrepo M., Gené J., Castañeda-Ruiz R., Mena-Portales J., Crous P., Guarro J. (2017). Phylogeny of saprobic microfungi from Southern Europe. Stud. Mycol..

[8] Hyde K.D., Dong Y., Phookamsak R., Jeewon R., Bhat D.J., Jones E.B.G., Liu N.-G., Abeywickrama P.D., Mapook A., Wei D. (2020). Fungal diversity notes 1151–1276: Taxonomic and phylogenetic contributions on genera and species of fungal taxa. Fungal Divers..

[9] Réblová M., Seifert K.A., Fournier J., Štěpánek V. (2012). Phylogenetic classification of *Pleurothecium* and *Pleurotheciella* gen. nov. and its dactylaria-like anamorph (Sordariomycetes) based on nuclear ribosomal and protein-coding genes. Mycologia.

[10] Luo Z.-L., Hyde K.D., Bhat D.J., Jeewon R., Maharachchikumbura S.S.N., Bao D.-F., Li W.-L., Su X.-J., Yang X.-Y., Su H.-Y. (2018). Morphological and molecular taxonomy of novel species Pleurotheciaceae from freshwater habitats in Yunnan, China. Mycol. Prog..

[11] Wijayawardene N.N., Hyde K.D., Al-ani L.K.T., Tedersoo L., Haelewaters D., Rajeshkumar K.C., Zhao R.L., Aptroot A., Leontyev D.V., Saxena R.K. (2020). Outline of Fungi and fungus-like taxa. Mycosphere.

[12] Hyde K.D., Norphanphoun C., Abreu V.P., Bazzicalupo A., Chethana K.W.T., Clericuzio M., Dayarathne M.C., Dissanayake A.J., Ekanayaka A.H., He M.-Q. (2017). Fungal diversity notes 603–708: Taxonomic and phylogenetic notes on genera and species. Fungal Divers..

[13] Hyde K.D., Chaiwan N., Norphanphoun C., Boonmee S., Camporesi E., Chethana K.W.T., Dayarathne M.C., de Silva N.I., Dissanayake A.J., Ekanayaka A.H. (2018). Mycosphere notes 169–224. Mycosphere.

[14] Su H.-Y., Udayanga D., Luo Z.-L., Manamgoda D., Zhao Y.-C., Yang J., Liu X.-Y., McKenzie E., Zhou D.-Q., Hyde K. (2015). Hyphomycetes from aquatic habitats in Southern China: Species of *Curvularia* (Pleosporaceae) and *Phragmocephala* (Melannomataceae). Phytotaxa.

[15] Fallah P.M., Crane J.L., A Shearer C. (1999). Freshwater ascomycetes: Two new species of *Ascotaiwania* from North America. Can. J. Bot..

[16] Fernández F.A., Lutzoni F.M., Huhndorf S.M. (1999). Teleomorph-anamorph connections: The new pyrenomycetous genus *Carpoligna* and its *Pleurothecium* anamorph. Mycologia.

[17] Chomnunti P., Hongsanan S., Aguirre-Hudson B., Tian Q., Persoh D., Dhami M.K., Alias A.S., Xu J., Liu X., Stadler M. (2014). The sooty moulds. Fungal Divers..

[18] Senanayake I.C., Rathnayaka A.R., Marasinghe D.S., Calabon M.S., Gentekaki E., Lee H.B., Hurdeal V.G., Pem D., Dissanayake L.S., Wijesinghe S.N. (2020). Morphological approaches in studying fungi: Collection, examination, isolation, sporulation and preservation. Mycosphere.

[19] Jayasiri S.C., Hyde K.D., Ariyawansa H., Bhat J., Buyck B., Cai L., Dai Y.-C., Abd-Elsalam K.A., Ertz D., Hidayat I. (2015). The Faces of Fungi database: Fungal names linked with morphology, phylogeny and human impacts. Fungal Divers..

[20] Vilgalys R., Hester M. (1990). Rapid genetic identification and mapping of enzymatically amplified ribosomal DNA from several *Cryptococcus* species. J. Bacteriol..

[21] White T.J., Bruns T., Lee S., Taylor J., Innis M.A., Gelfand D.H., Sninsky J.J., White T.J. (1990). Amplification and Direct Sequencing of Fungal Ribosomal RNA Genes for Phylogenetics. PCR Protocols: A Guide to Methods and Applications.

[22] Liu Y.J., Whelen S., Hall B.D. (1999). Phylogenetic relationships among ascomycetes: Evidence from an RNA polymerse II subunit. Mol. Biol. Evol..

[23] Dong W., Hyde K.D., Doilom M., Yu X.-D., Bhat D.J., Jeewon R., Boonmee S., Wang G.-N., Nalumpang S., Zhang H. (2020). *Pseudobactrodesmium* (Dactylosporaceae, Eurotiomycetes, Fungi) a Novel Lignicolous Genus. Front. Microbiol..

[24] Dong W., Zhang H., Doilom M., Yu X.-D., Wang G.-N., Nalumpang S. (2021). *Rhexodenticula aquatica* (Sordariomycetidae genera *incertae sedis*), a novel hyphomycete from freshwater in Thailand. Phytotaxa.

[25] Katoh K., Standley D.M. (2016). A simple method to control over-alignment in the MAFFT multiple sequence alignment program. Bioinformatics.

[26] Miller M., Pfeiffer W., Schwartz T. Creating the CIPRES Science Gateway for inference of large phylogenetic trees. Proceedings of the Gateway Computing Environments Workshop.

[27] Miller M.A., Schwartz T., Pickett B., He S., Klem E.B., Scheuermann R.H., Passarotti M., Kaufman S., O’Leary M.A. (2015). A RESTful API for Access to Phylogenetic Tools via the CIPRES Science Gateway. Evol. Bioinform..

[28] Larget B., Simon D.L. (1999). Markov Chasin Monte Carlo Algorithms for the Bayesian Analysis of Phylogenetic Trees. Mol. Biol. Evol..

[29] Doilom M., Dissanayake A.J., Wanasinghe D., Boonmee S., Liu J.-K., Bhat D.J., Taylor J.E., Bahkali A.H., McKenzie E.H.C., Hyde K.D. (2017). Microfungi on *Tectona grandis* (teak) in Northern Thailand. Fungal Divers..

[30] Charles V.K. (1929). *Coledictyospora*, a new genus of dematiaceae. Phytopathology.

[31] Matsushima T. (1987). Matsushima mycological memoirs n. 5. Matsushima Fungus: Kobe.

[32] Fryar S., Davies J., Booth W., Hodgkiss I., Hyde K. (2004). Succession of fungi on dead and live wood in brackish water in Brunei. Mycologia.

[33] Nakagiri A., Ito T. (1995). Some dematiaceous hyphomycetes on decomposing leaves of *Satakentia liukiuensis* from Ishigaki Island, Japan. IFO Res. Comm..

[34] Ho W.H., Yanna, Hyde K.D., Hodgkiss I.J. (2002). Seasonality and sequential occurrence of fungi on wood submerged in Tai Po Kau Forest Stream, Hong Kong. Fungal Divers..

[35] Hernández A., Mena J. (1995). Hifomicetos asociados a *Coccothrinax* (Palmae) en diferentes localidades de la Provincia de Camagüey (Cuba). Bol. Soc. Micol. Madr..

[36] Delgado G. (2009). South Florida microfungi: Veramycella bispora, a new palmicolous anamorphic genus and species, with some new records for the continental USA. Mycotaxon.

[37] Whitton S.R., McKenzie E.H.C., Hyde K.D., Whitton S.R., McKenzie E.H.C., Hyde K.D. (2012). Anamorphic fungi associated with Pandanaceae. Fungi Associated with Pandanaceae.

[38] Becerra Hernández C.I., Heredia G., Arias R.M., Mena Portales J., Castañeda Ruiz R.F. (2008). Los hongos anamorfos saprobios del estado de Tabasco. III. Rev. Mex. Micol..

[39] Matsushima T. (1981). Matsushima mycological memories n. 2. Matsushima Fungus: Kobe.

[40] Matsushima T. (1993). Matsushima mycological memories n. 7. Matsushima Fungus: Kobe.

[41] Pinnoi A., Lumyong S., Hyde K.D., Jones E.B.G. (2006). Biodiversity of fungi on the palm *Eleiodoxa conferta* in Sirindhorn peat swamp forest, Narathiwat, Thailand. Fungal Divers..

[42] Seifert K.A., Morgan-Jones G., Gams W., Kendrick B. (2011). The genera of Hyphomycetes.

[43] Sun L.Y., Li H.Y., Sun X., Guo L.D. (2017). *Dematipyriforma aquilaria* gen. et sp. nov., a new hyphomycetous taxon from *Aquilaria crassna*. Cryptogam. Mycol..

[44] Baker W.A., Partridge E.C., Morgan-Jones G. (2002). Notes on hyphomycetes LXXXVII. *Rhexoacrodictys*, a new segregate genus to accommodate four species previously classified in *Acrodictys*. Mycotaxon.

[45] Xiao Z.-J., Li X.-X., Wang H.-D., Song P.-Y., Tang L. (2018). *Rhexoacrodictys broussonetiae* sp. nov. from Guizhou, China. Mycotaxon.

[46] Sivanesan A., Chang H. (1992). *Ascotaiwania*, a new amphisphaeriaceous ascomycete genus on wood from Taiwan. Mycol. Res..

[47] Chang H., Hsieh S.-Y., Jones E., Read S., Moss S. (1998). New freshwater species of *Ascotaiwania* and *Savoryella* from Taiwan. Mycol. Res..

[48] Dong W., Wang B., Hyde K.D., McKenzie E.H.C., Raja H.A., Tanaka K., Abdel-Wahab M.A., Abdel-Aziz F.A., Doilom M., Phookamsak R. (2020). Freshwater Dothideomycetes. Fungal Divers..

[49] Arzanlou M., Groenewald J., Gams W., Braun U., Shin H.-D., Crous P. (2007). Phylogenetic and morphotaxonomic revision of *Ramichloridium* and allied genera. Stud. Mycol..

[50] Boonmee S., Rossman A.Y., Liu J.-K., Li W.-J., Dai D.-Q., Bhat J.D., Jones E.B.G., McKenzie E.H.C., Xu J.-C., Hyde K.D. (2014). Tubeufiales, ord. nov., integrating sexual and asexual generic names. Fungal Divers..

[51] Lu Y.-Z., Liu J.-K., Hyde K.D., Jeewon R., Kang J.-C., Fan C., Boonmee S., Bhat D.J., Luo Z.-L., Lin C.-G. (2018). A taxonomic reassessment of Tubeufiales based on multi-locus phylogeny and morphology. Fungal Divers..

[52] Kodsueb R., Jeewon R., Vijaykrishna D., McKenzie E.H.C., Lumyong P., Lumyong S., Hyde K.D. (2006). Systematic revision of Tubeufiaceae based on morphological and molecular data. Fungal Divers..

[53] Zhang H., Dong W., Hyde K.D., Maharachchikumbura S., Hongsanan S., Bhat D.J., Al-Sadi A., Zhang D. (2017). Towards a natural classification of Annulatascaceae-like taxa: Introducing Atractosporales ord. nov. and six new families. Fungal Divers..

[54] Dong W., Hyde K.D., Jeewon R., Doilom M., Yu X.D., Wang G.N., Liu N.G., Hu D.M., Nalumpang S., Zhang H. (2021). Towards a natural classification of annulatascaceae-like taxa II: Introducing five new genera and eighteen new species from freshwater. Mycosphere.

[55] Jeewon R., Hyde K.D. (2016). Establishing species boundaries and new taxa among fungi: Recommendations to resolve taxonomic ambiguities. Mycosphere.

[56] Xia J.W., Ma Y.R., Li Z., Zhang X.G. (2017). Acrodictys-like wood decay fungi from southern China, with two new families Acrodictyaceae and Junewangiaceae. Sci. Rep..

[57] Bucher V., Pointing S., Hyde K., Reddy C. (2004). Production of Wood Decay Enzymes, Loss of Mass, and Lignin Solubilization in Wood by Diverse Tropical Freshwater Fungi. Microb. Ecol..

[58] Hyde K.D., Fryar S., Tian Q., Bahkali A.H., Xu J. (2016). Lignicolous freshwater fungi along a north–south latitudinal gradient in the Asian/Australian region; can we predict the impact of global warming on biodiversity and function?. Fungal Ecol..

[59] Fryar S.C., Booth W., Davies J., Hodgkiss I.J., Hyde K.D. (2004). Distribution of fungi on wood in the Tutong River, Brunei. Fungal Divers..

[60] Kodsueb R., Lumyong S., McKenzie E., Bahkali A., Hyde K. (2016). Relationships between terrestrial and freshwater lignicolous fungi. Fungal Ecol..

[61] Hyde K.D., Jeewon R., Chen Y.-J., Bhunjun C.S., Calabon M.S., Jiang H.-B., Lin C.-G., Norphanphoun C., Sysouphanthong P., Pem D. (2020). The numbers of fungi: Is the descriptive curve flattening?. Fungal Divers..

[62] Hyde K.D., Norphanphoun C., Maharachchikumbura S.S.N., Bhat D.J., Jones E.B.G., Bundhun D., Chen Y.J., Bao D.F., Boonmee S., Calabon M.S. (2020). Refined families of Sordariomycetes. Mycosphere.

[63] Ho W.H., Hyde K.D., Hodgkiss J.I. (2001). Fungal communities on submerged wood from streams in Brunei, Hong Kong, and Malaysia. Mycol. Res..

