# Supplementary figures and images for: Five Novel Taxa from Freshwater Habitats and New Taxonomic Insights of Pleurotheciales and Savoryellomycetidae
Source: J Fungi (Basel). 2021 Aug 30;7(9):711. doi: 10.3390/jof7090711 (PMC8470061; doi:10.3390/jof7090711)

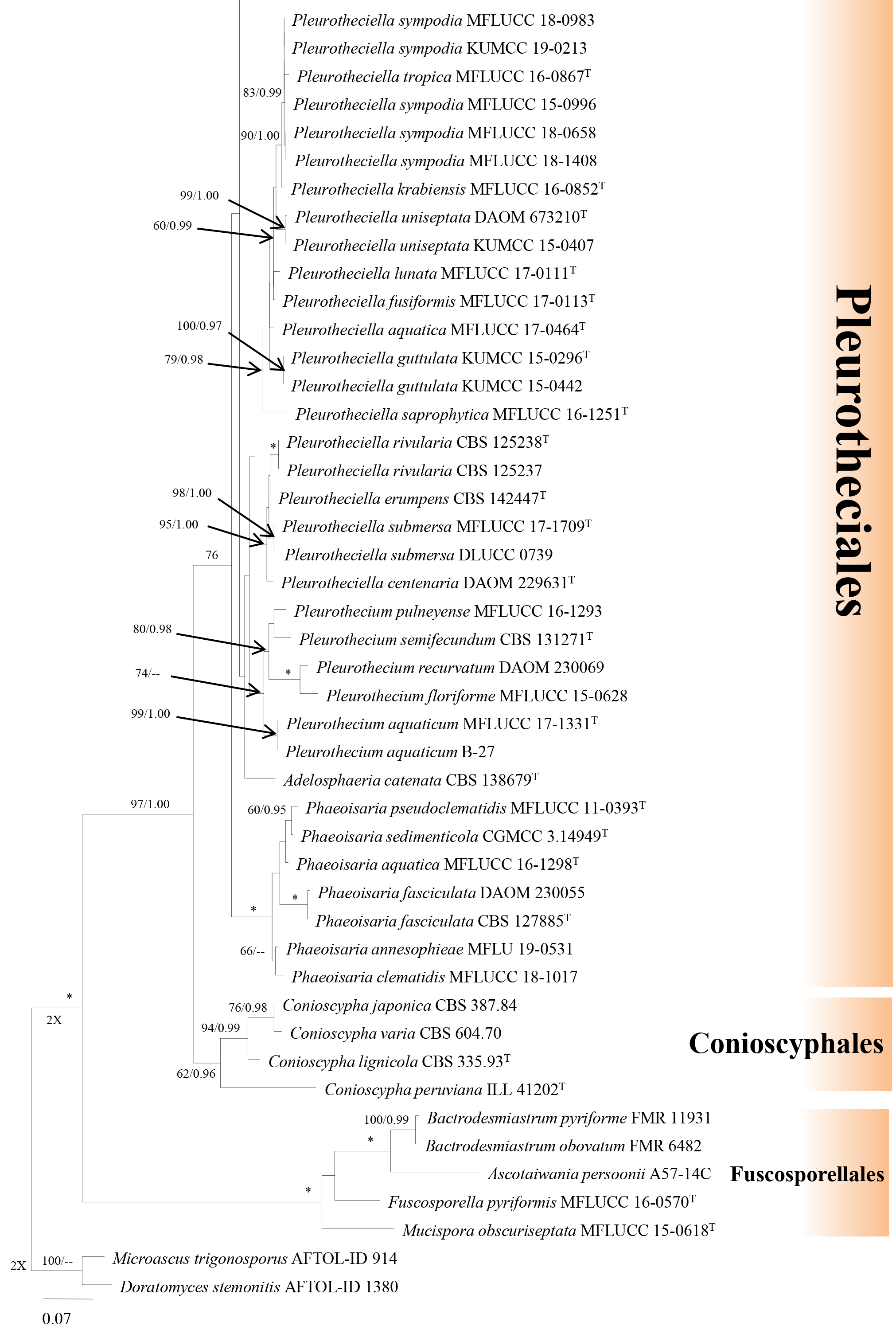

Supplement: Supplementary file 1 [file jof-07-00711-s001.zip › Figure S1 Continued.jpg]

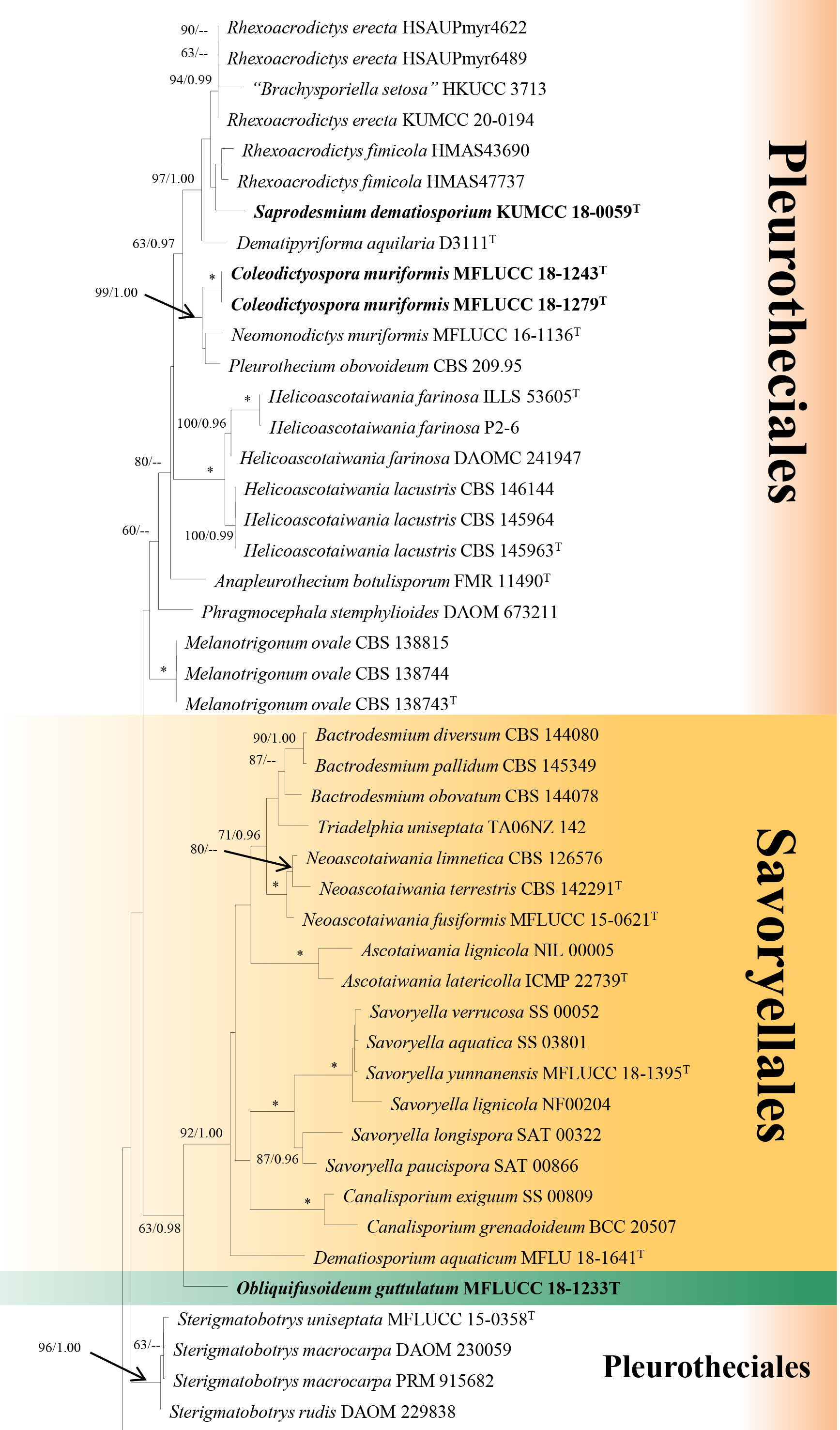

Supplement: Supplementary file 1 [file jof-07-00711-s001.zip › Figure S1.jpg]

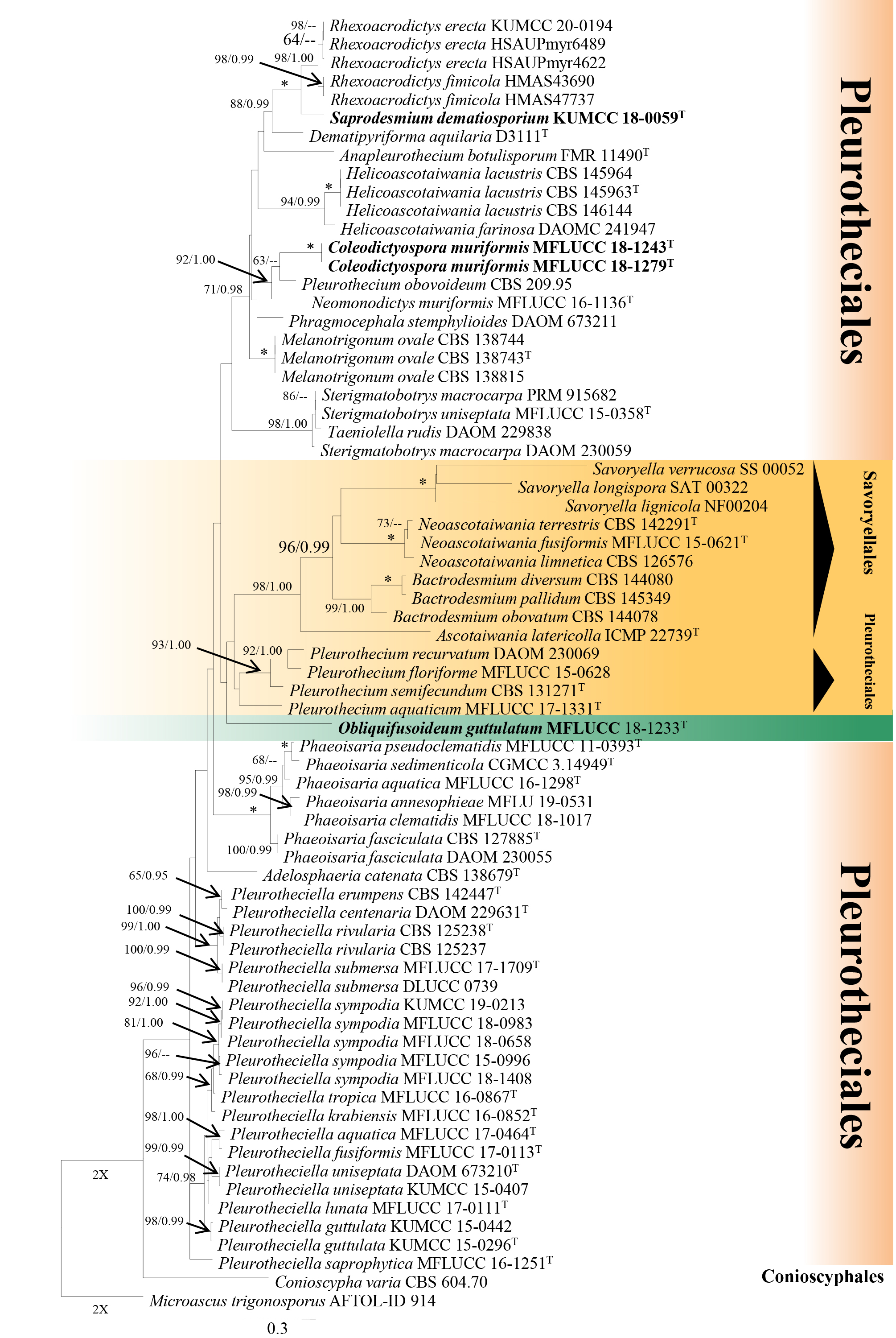

Supplement: Supplementary file 1 [file jof-07-00711-s001.zip › Figure S2.jpg]
